# An assessment of seismicity and near surface geophysical characteristics of potential solid waste landfill sites in the Eastern Black Sea Region of Türkiye

**DOI:** 10.1007/s11356-024-31964-4

**Published:** 2024-01-26

**Authors:** Hakan Karslı, Ali Erden Babacan, Nilgün Sayıl, Kaan Hakan Çoban, Özgenç Akın

**Affiliations:** Department of Geophysics, Karadeniz Technical University, Trabzon, Türkiye

**Keywords:** Solid waste landfill, Eastern Black Sea Region, Seismicity, SRT, MASW, ERT

## Abstract

This study aimed to assess the suitability of the potential solid waste landfill sites in seven provinces (Samsun, Ordu, Giresun, Trabzon, Gümüşhane, Bayburt and Artvin) in the Eastern Blacksea Region of Türkiye. The earthquake hazard analysis for two major earthquakes which occurred in the region was first carried out. Then, the geophysical methods including seismic refraction tomography (SRT), electrical resistivity tomography (ERT) and Multichannel Analysis of Surface Waves (MASW) were conducted to find out the structural and physical properties of the subsurface which include the layering, soil classification based on *V*_*S*30_ and the groundwater content at 25 locations of 13 in target provinces. The integrated interpretation of whole data sets demonstrates that Işıktepe, Esence, Çamburnu and Kazantaş which are characterized by *V*_*P*_ > 1200 m/s, *V*_*S*30_ ≥ 400 m/s, *ρ* > 70 Ohm-m, low earthquake hazard and seismicity are more suitable among others. Vezirköprü, Şebinkarahisar, Yenice, Bayburt-Center, Balkaynak and Murgul will be suitable after a geotechnical reclamation due to moderate seismic velocities and electrical resistivity which are 900 < *V*_*P*_ ≤ 1200 m/s, 200 < *V*_*S*30_ < 400 m/s and 10 < *ρ* ≤ 70 Ohm-m representing stiff and wet soils. In addition, Bafra, Ağalık and Ovacık were considered to be unsuitable due to the presence of thick, water-saturated soft soil and extremely weathered rocks. Finally, this study shows that the joint interpretation of seismicity and geophysical data in potential waste landfill sites, extremely important for the planning and development of a city, can provide the valuable information which will enable to prevent possible deformations, environmental problems and economic losses after waste landfill.

## Introduction

In many countries, due to the rapidly increasing population and industrial facilities, uncontrolled growth and expansion of new urban areas, the production of solid waste (domestic and/or industrial waste) brings problems with its landfill and disposal. In recent years, pollution of the underground water resources and the environment, especially by leachate from old and existing irregular and uncontrolled landfills, landslides and collapses in the dumping areas have posed a high risk to public health (EPA 1999; Özel et al. 2017). On the other hand, with the development of new-generation construction and manufacturing technologies, sanitary landfills and disposal sites are now built with appropriate procedures according to engineering standards and then they are constantly monitored and inspected by local governments and environmental agencies. However, inadequacies in the construction and operation of many landfills still remain relevant in small and medium-sized cities, particularly regarding existing but deactivated landfills (Soupios et al. 2007). The fact that most of the waste landfill areas are close to settlements, markets, farms, highways, industrial areas, etc. has revealed the necessity of carefully examining not only the environmental conditions but also the soil and rock characteristics of the selected places before landfilling. Therefore, the demand for examining the suitability of the sites considered for future landfill planning in terms of environmental and structural characteristics has increased significantly in the last few years due to the lack of space in dense settlements (Rao 1997; Dorhofer and Siebert 1998; Dorn and Tantiwanit 2001; Mondelli et al. 2012). For this reason, the problems of environmental pollution and waste management have become a major area of interest for geoscientists and researchers from other related sciences.

Currently, in international and national regulations, preliminary studies in terms of city planning, geological, hydrogeological, geophysical, environmental science, social and health sciences and geographic information systems have become mandatory for the selection, design and construction of suitable solid waste landfill areas (Choudhury and Savoikar 2009). In addition to knowing the local geological characteristics of potential waste landfill areas, it is necessary to know their geophysical characteristics in detail. On the other hand, alternative areas for solid waste landfill should be evaluated specially in terms of seismology, their distance to active faults, historical and instrumental seismicity and the earthquake characteristics (magnitudes and earthquake recurrence times) (Krinitzsky et al. 1997; Yeşilnaçar and Çetin 2005). Moreover, earthquake hazard and risk of the region should be explained according to scientific methods.

Although the mechanical methods (standard penetration, cone penetration, pressure metre tests, etc.) used for subsurface characterization of an area provide reliable information about soil thickness and type, these tests are costly (money, labour and time) and destructive (Kosugi et al. 2006). However, the main disadvantage of mechanical methods is that they provide information for subsurface soil characteristics from only one investigation point and therefore require many exploration drillings to determine complex geological structures or lateral facies changes. The main way to overcome this problem is to carry out geophysical investigations. Because geophysical methods help reduce the number of these traditional and more expensive mechanical investigations, they make a significant contribution to directly reducing project costs (Zhan et al. 2018; Boudreault et al. 2010; Fernández-Baniela et al. 2021).

Geophysical methods, which allow rapid exploration of large areas without changing their natural conditions at each site in depth and spatial dimensions, allow the non-invasive determination of physical properties and mechanical behaviour of the soils and rocks such as softness-stiffness characterization of the geological units, soil classification, possible structural elements (faults, fractures, voids, caves, cracks, etc.), geometry of the soil (soil thickness and depth to the bedrock), dynamic-elastic parameters and groundwater content. Accordingly, geophysical engineering studies have recently become an important area of use in the waste management workflow, and the use of geophysical methods has become widespread (Otto and Sass 2006; Schrott and Sass 2008; Kowalczyk et al. 2017). In this context, magnetic, electrical resistivity, electromagnetic, natural potential, ground radar penetrating and seismic (refraction, reflection and surface wave analysis) methods are frequently applied. Thus, the geophysical methods include providing the information about the subsurface distribution of certain physical parameters (density, seismic velocity, electrical resistivity and conductivity, magnetic susceptibility, dielectric permeability and electromagnetic velocity, etc.). Furthermore, the combined use of geophysical methods provides important opportunities for the evaluation and characterization of the pollution caused by solid wastes (Kayabalı 1996; Önal et al. 2013; Konstantaki et al. 2015; Çınar et al. 2016). For this reason, possible soil problems such as sliding, collapse, breaking and cracking in waste landfill areas, mixing of waste leachate with groundwater and polluting water resources, damage to the natural built environment and related health and socio-economic damages will be prevented (Tchobanoglous and Theisen 1993; Pomposiello et al. 2012).

Although geophysical methods have a wide range of applications, their use in detecting and monitoring of the waste and solving the problems arising from landfill areas is methodologically limited. Moreover, when the literature sources are examined, the reasons for using and applying geophysical methods for solid waste landfills can be grouped into two main groups:

1. In the past and up to the present, the first group of studies has mostly involved in monitoring the existing landfills, types of the old waste and their distribution, delimitating the waste dump area, detecting and solving the problems (leakage, collapse, outgassing, etc.) by using geophysical methods (Siracusa et al. 2005; Kaya et al. 2007; Balia and Littarru 2010; Genelle et al. 2012; Özel 2018; Dumont et al. 2018; Neyamadpour 2019).
2. The second group of studies was the study that led to the final decision about the suitability of the potential and/or alternative waste landfill area. They include detailed examination of seismicity of the region, flood, landslide hazards-risks and soil characteristics after preliminary studies which use the remote sensing methods for the selection of landfill area location (Reynolds and Taylor 1996; Silvestri and Omri 2008; Mondelli et al. 2012; Pomposiello et al. 2012; Soupios and Ntarlagiannis 2017; El-Kelani and Khader 2019). Thus, these studies, which include near surface geophysical methods, seismic hazard and risk assessments, have recently started to be preferred in the workflow where site selection decisions are made.

In this study, within the framework of previously mentioned second group of studies, we firstly overviewed the seismicity of the region and then we indicated an integration of geophysical (SRT, MASW, ERT) methods in 25 profiles to characterize subsoil materials and their potentially risks for waste landfill site assessments for seven provinces (Samsun, Ordu, Giresun, Trabzon, Gümüşhane, Bayburt and Artvin) in the Eastern Black Sea of Türkiye. This is demonstrated in terms of investigating the subsurface materials within the upper, approximately 30-m-thick layer, examining the near surface/bedrock structure under landfills such as near-surface bedding, softness-stiffness of soils and low–high resistive zones and evaluating the suitability of the studied areas by geophysical data.

## Study area and surveying locations

**Fig. 1:**
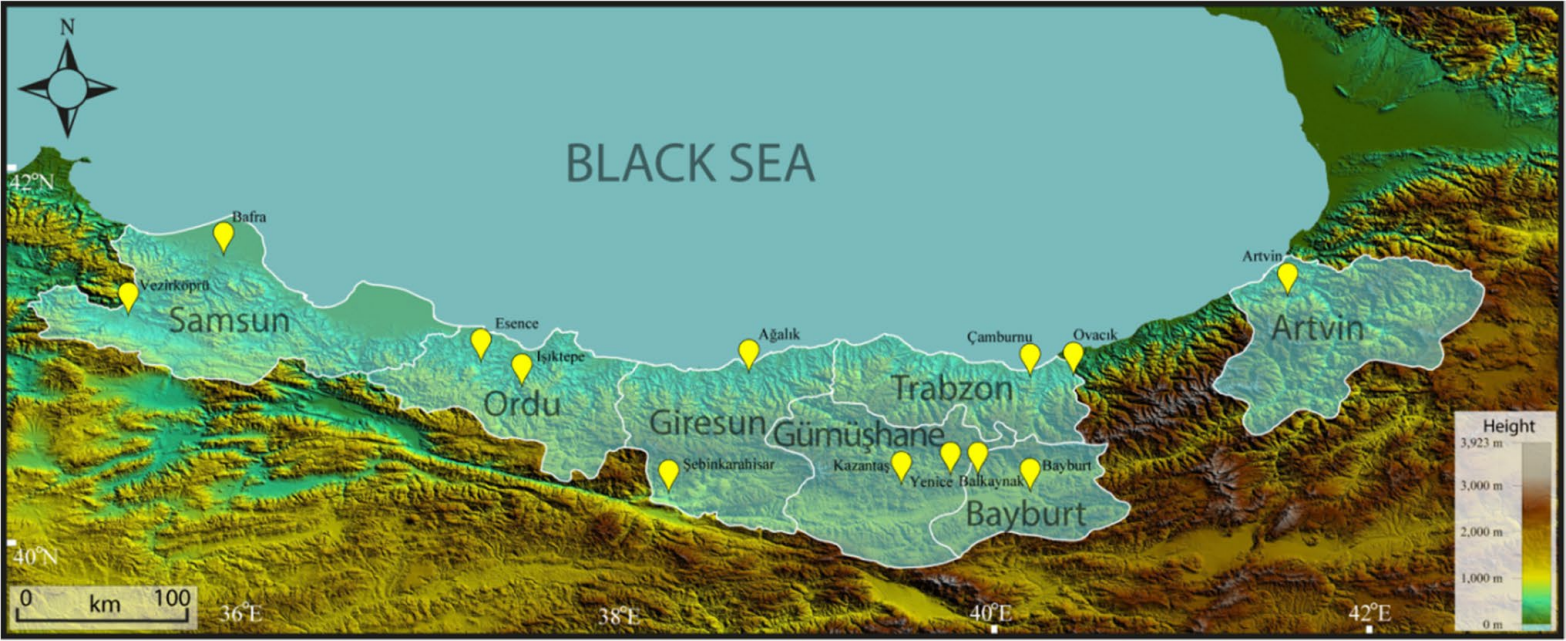
The provinces where landfill areas will be determined and the selected geophysical (SRT, MASW, ERT) measurement locations (yellow pins)

The study area includes provinces (Samsun, Ordu, Giresun, Trabzon, Gümüşhane, Bayburt and Artvin) in the area of responsibility of the Eastern Black Sea Project Regional Development Administration (DOKAP), which covers an area of approximately 49,000 km^2^ in the Eastern Black Sea Region and operates to accelerate the development of the region (Fig. 1). The total population of these provinces is approximately 4,000,000 people. According to Yıldırım et al. (2022), in the DOKAP (2021) report, it is stated that, as of 2018, the largest amount of waste was dumped in Samsun (~ 402,000 tons), and the smallest amount of them was disposed in Bayburt (~ 16,000 tons). The province with the largest average amount of municipal waste was Rize (1.25 kg/day/person), and the province with the smallest average amount of municipal waste was Trabzon (0.81 kg/day/person) (DOKAP 2021).

However, according to preliminary studies (environmental and sociological criteria in accordance with national and international legislation) which include the opinions of institutions, organizations and external stakeholders in Rize Municipality, no potential suitable landfill area could be determined. Therefore, seismicity and near-surface geophysical studies were not carried out for this municipal.

### Regional geology

**Fig. 2:**
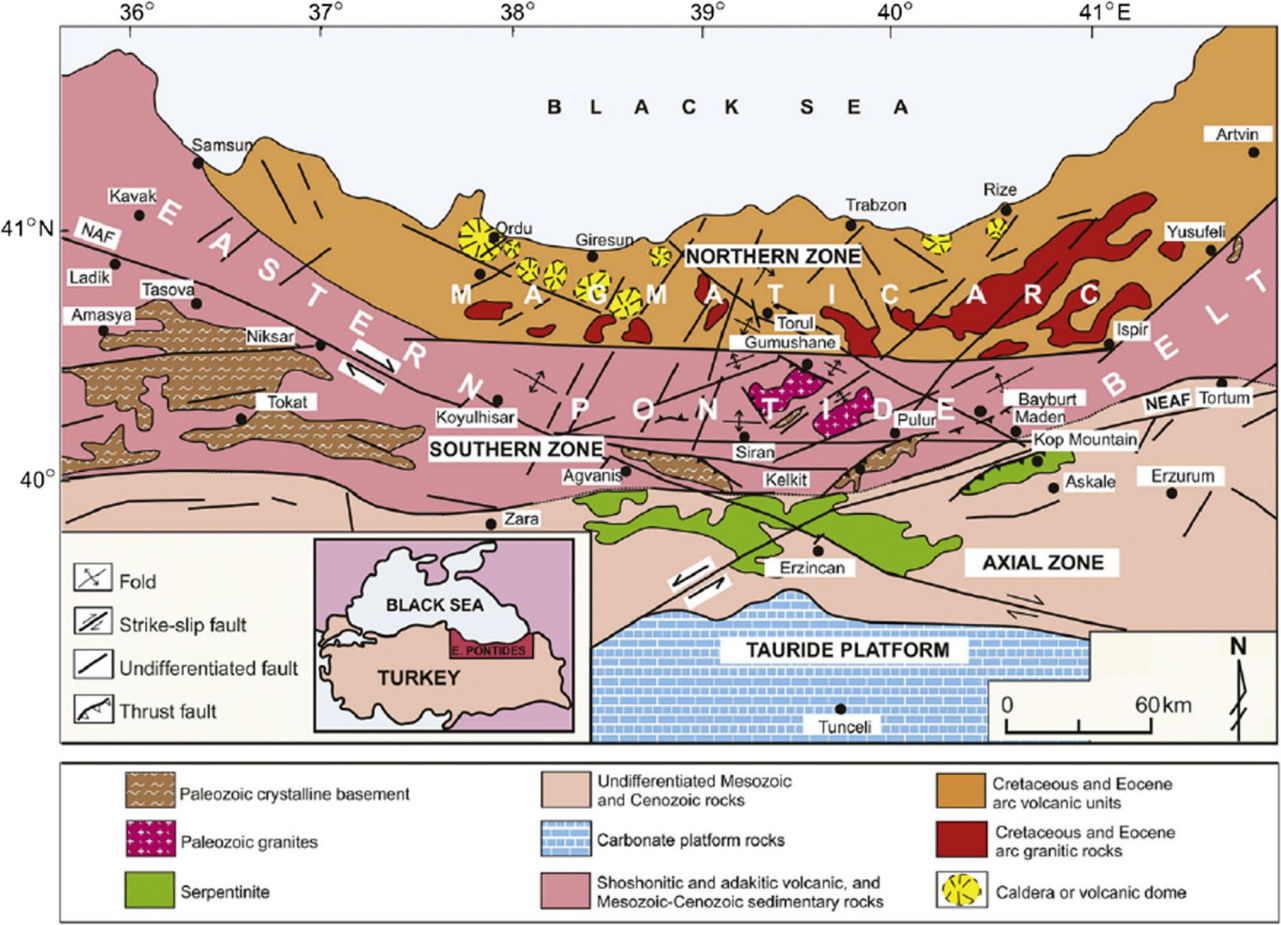
Main geological and tectonic features of the Eastern Pontide Orogenic Belt. NAF, North Anatolian Fault; NEAF, Northeast Anatolian Fault (from Eyüboğlu et al. 2011)

The Eastern Black Sea Region is located in the north-eastern part of Türkiye which consists a mountain chain approximately 500 km long and 200 km wide parallel to the southeast coast of the Black Sea and is located on the Eastern Pontide Orogenic Belt tectonic unit (Ketin 1976). As shown in Fig. 2, this tectonic unit is divided into three different subunits depending on lithological differences, geological and geophysical features, tectonic structures and lithological changes from north to south: the north zone, south zone and axis zone (Bektaş et al. 1995; Eyüboğlu et al. 2011). The paleo-tectonic evolution of the Eastern Pontide Orogenic Belt was controlled by faults of three different directions. Because the small and large blocks (north, south and axis zone) formed by the faults move independently and relatively, the geological features of each block are different (Eyüboğlu et al. 2011). Folds that develop because of the horizontal and vertical movements of the blocks have the characteristics of drag or drape folds parallel or almost parallel to the block edges. While Mesozoic and Cenozoic aged volcanic rocks and granitic intrusions are generally dominant in the northern zone, sedimentary rock series constitute the dominant lithology in the Southern Zone. Güven (1993), who conducted one of the most detailed studies in the region, examined the succession in the northern zone from the bottom to the top and named all units as the formation and lithology. According to Güven (1993), volcano-sedimentary series and volcanic and intrusive rocks containing the products of magmatism that continued their development from the Early Jurassic until the end of the Eocene are common in the region where rock units developed in the Paleozoic-Quaternary period are exposed. Moreover, sedimentary successions accumulated during periods when magmatic activity stopped.

**Fig. 3:**
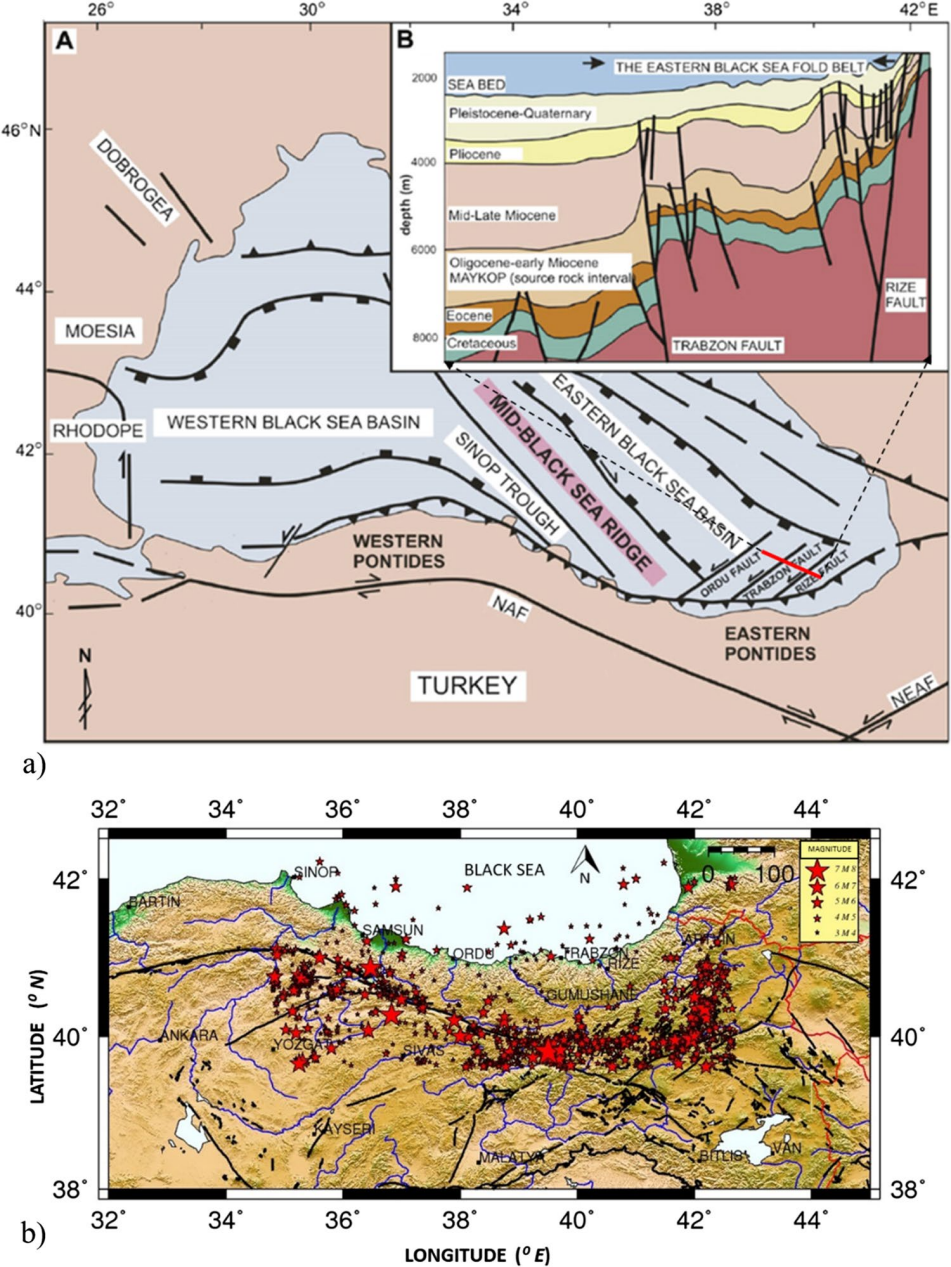
Main faults and seismicity of Eastern Black Sea Region. **a** Faults in the land and offshore threatening the region (adapted from Eyüboğlu et al. 2011). Red thick line in (A) approximately indicates the location of the cross section in (B). **b** Epicentral distributions of earthquakes with *M*_*s*_ ≥ 3.0 (1900–2016) in the region. Faults are digitized by using Emre et al. (2013)

Türkiye is an active and complex region in which the Eurasian, Arabian and Anatolian plates meet (McKenzie 1972; Alptekin 1973; Ketin 1977). Consequently, the Anatolian plate moves westward between the dextral strike-slip North Anatolian Fault zone (NAFZ) and the left-lateral strike-slip Eastern Anatolian Fault zone (EAFZ). In this system, as shown in Fig. 3a, the region is under the influence of a thrust fault (inclined to the south) parallel to the coast in the north (Okay and Sahintürk 1997; Nikishin et al. 2003) and NE-SW extension strike-slip faults called Rize, Trabzon and Ordu in marine side, which have been determined based on geological, geophysical and GPS data collected for the last 30 years (Eyüboğlu et al. 2011; Meisner et al. 2009; Keskin et al. 2010).

## Material and methods

### Seismicity studies

Historical (Ambraseys and Jacson 1981; Gündoğdu and Altınok 1986; Ayhan et al. 1987) and instrumental data on earthquake occurred in the region that may affect potential waste landfill sites, national (Boğaziçi University Kandilli Observatory-KOERI) and international (International Seismological Center-ISC) were provided by the catalogues published by the US Geological Survey-USGS data centres. Different magnitudes (*M*_*b*_, *M*_*L*_, *M*_*d*_, *M*_*w*_) in the catalogues of the instrumental period between 1900 and 2016 were converted to surface wave magnitude (*M*_*s*_) by using the transformation equations for Türkiye and its surroundings given by Aydın (2016), and the catalogue was homogenized. In addition, the intensity scale describing historical earthquakes was transformed into a magnitude scale using the Eq. (1), developed by Sayıl (2014).

1 $${M}_{s}=0.47\left(\pm {\sigma }_{1}\right){I}_{o}+2.05\left(\pm {\sigma }_{2}\right)$$

Here, *I*_*o*_, *σ*_1_ =  ± 0.02 and *σ*_2_ =  ± 0.24 indicate the earthquake intensity and errors in the least square linear regression approach, respectively. Thus, the magnitude-epicentre distribution for the lower limit *M*_*s*_ ≥ 4.0 of the data set obtained by applying the completeness analysis of the earthquake data is shown in Fig. 3b. Most of the earthquake locations shown in Fig. 3b occurred in the NAFZ. However, the offshore earthquakes are thought to be related to the coastal parallel thrust fault shown in Fig. 3a and the strike-slip Rize, Trabzon and Ordu faults. Readers can find detailed information from Eyüboğlu et al. (2011). The total length of the North Anatolian Fault Zone (NAFZ), which is a right-hand strike-slip fault system, is approximately 1500 km. While the eastern part of the fault was under compression, the western part extended. The focal mechanisms of earthquakes occurring so far along the NAFZ confirm these different stress regimes (Sayıl 2014). The major historical (*M*_*s*_ ≥ 7.0) earthquakes along the NAFZ are listed in Table 1.

**Table 1:** The large historical earthquakes (*M*_*s*_ ≥ 7.0) in the Eastern Black Sea Region (KOERI)

Date	Hour	Latitude (°N)	Longitude (°E)	Intensity	Location
1045		39.75	39.50	IX	Erzincan
1268		39.75	40.40	IX	Erzincan, Erzurum-(15,000 dead)
1458		39.75	40.40	X	Erzincan, Erzurum-(32,000 dead)
21 Dec 1482		39.75	39.50	IX	Erzincan, Erzurum
17 Jun 1584		39.75	39.50	IX	Erzincan, Erzurum-(15,000 dead)
24 Jul 1852		39.90	41.30	IX	Erzurum
02 Jun 1859	10:30	39.90	41.30	IX	Erzurum-(15,000 dead)
23 Apr 1868		40.00	41.70	IX	Erzurum, Kars
01 Nov 1875	10:00	39.90	41.30	X	Erzurum
20 May 1890		39.90	38.80	IX	Refahiye, Erzincan

In the NAFZ, the 1939 (*M*_*s*_ = 7.9) and 1992 (*M*_*s*_ = 6.6) earthquakes and the 1942 (*M*_*s*_ = 7.0) earthquake occurred in Erzincan causing significant loss of life and property by affecting seven municipalities covering the study area.

**Fig. 4:**
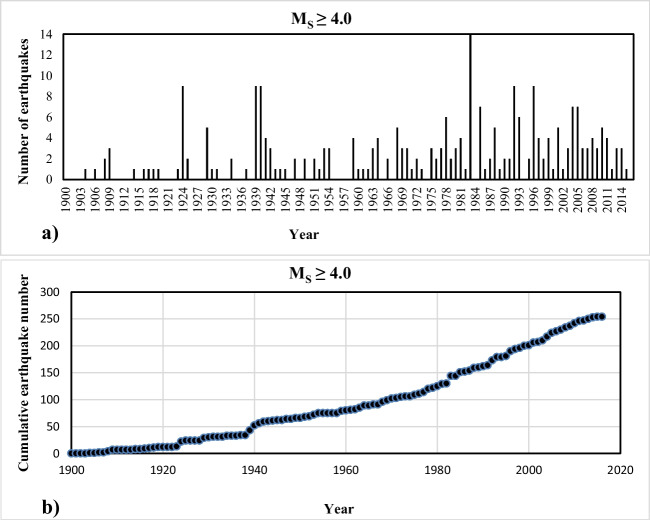
Earthquakes statistics analysed within the scope of the study. **a** Annual. **b** Cumulative distributions of earthquakes with *M*_*s*_ ≥ 4.0

The NAFZ is a fault zone with a high seismicity. Therefore, the seismicity of the region was examined for the region and each province. The annual number of earthquakes with *M*_*s*_ ≥ 4.0 in the study area (histogram) is shown in Fig. 4a and the cumulative number of earthquakes is shown in Fig. 4b. Accordingly, 265 earthquake data were used, and the magnitude range was taken as ∆*m* = 0.1 to evaluate the Gutenberg-Richter magnitude-frequency relationship of the region.

For an earthquake with a magnitude of *M*_*s*_ ≥ 4.0 which may occur in the study area, the Poisson model was used to calculate the probability of an earthquake occurring in the past or in the future. The probability of the occurrence of “*n*” earthquakes with magnitude *M* ≥ *M*_0_ at time *t* in a region studied is given as:

2 $$Pr\left(N=n|\nu ,t\right)=\frac{{e}^{-\nu t}{\left(\nu t\right)}^{n}}{n!}$$

where *ν* is the average number of earthquakes per time unit (usually one year) in the studied region, and “*N*” is a variable showing the number of earthquakes in the region under study at time “*t*”.

Magnitude-frequency relations are the basis of earthquake statistics and provide valuable information about the earthquake activity. A relationship between occurrence frequencies and magnitudes of the earthquakes is generally formulated by Eq. (3) (Gutenberg and Richter 1954).

3 $${\text{Log}}\;N\left(M\right)=a-bM$$

Here, *N* is the cumulative number of earthquakes, and *M* is the magnitude. Parameters *a* and *b* are the constants of Eq. (3), while parameter *a* depends on the observation period, the width of the study area and the level of earthquake activity the *b* value, which is the slope of the linear relationship, provides important information about earthquake formation physics and tectonic structure. Therefore, the *b* value is used in earthquake prediction processes and the preparation of seismotectonic zoning maps (Wiemer and Wyss 1997; Papazachos 1999; Öztürk 2012).

Thus, when Eq. (3) is obtained for a region, the probability that any earthquake of magnitude *M* will occur in that region for an observation interval of *T* years is given by Eq. (4):

4 $$R\left(M\right)={e}^{-n(M)T}$$

and the recurrence period is calculated by Eq. (5):

5 $$Q=\frac{1}{n(M)}$$

For this study, the regional magnitude-frequency relationship was obtained as follows. However, the magnitude-frequency relations for seven provinces are given in Table 2.

**Table 2:** Magnitude-frequency relations for each province where the waste landfill location will be determined

Provinces	Magnitude-frequency relation	Municipal	Magnitude-frequency relation
Samsun	Log *N*(*M*_*s*_) = 4.25–0.65 *M*_*s*_	Artvin	Log *N*(*M*_*s*_) = 5.06–0.87 *M*_*s*_
Ordu	Log *N*(*M*_*s*_) = 3.14–0.47 *M*_*s*_	Gümüşhane	Log *N*(*M*_*s*_) = 3.50–0.48 *M*_*s*_
Giresun	Log *N*(*M*_*s*_) = 3.83–0.57 *M*_*s*_	Bayburt	Log *N*(*M*_*s*_) = 4.33–0.68 *M*_*s*_
Trabzon	Log *N*(*M*_*s*_) = 2.40–0.48 *M*_*s*_		

6 $$\mathrm{Log\;}N\left(M\right)=5.3-0.76 M, \left(M={M}_{a}, {R}^{2}=0.98\right)$$

Here, *N*(*M*) represents the cumulative number of earthquakes. The parameters, *a* = 5.3, *b* = 0.76 and *R*^2^ are the correlation coefficient of the regression.

**Fig. 5:**
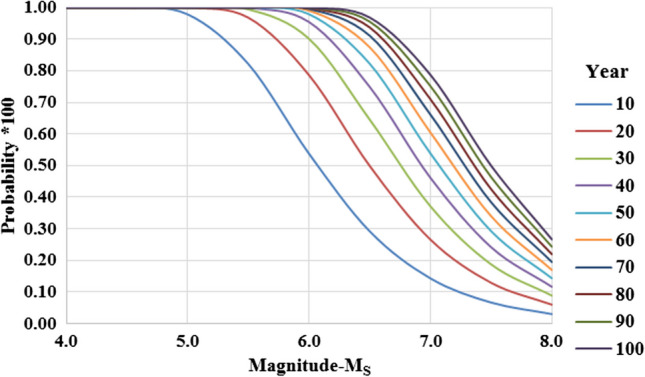
The occurrence probability of earthquakes in 10-year periods for different magnitudes in the region

Assuming the earthquakes occurring between 1900 and 2016 for the study region fit the Poisson distribution, probability calculations for earthquakes of various magnitudes were made by using Eq. (6). Accordingly, the probability of exceeding the earthquakes in every 10-year in the next 100 years, *R*(*M*) and recurrence periods (*Q*) were calculated and are given in Table 3. In addition, the probability of occurrence of earthquakes (hazards) in 10-year periods is shown graphically in Fig. 5.

**Table 3:** The probability of an earthquake with a magnitude of *M*_*s*_ ≥ 4.0 in the study area and its recurrence period values

Magnitude (*M*_*s*_)	Seismic hazard, *R*(*M*)% period (year)										Recurrence period (*Q*) year
	10	20	30	40	50	60	70	80	90	100	
4.0	100	100	100	100	100	100	100	100	100	100	0.5
4.5	100	100	100	100	100	100	100	100	100	100	1.2
5.0	98	100	100	100	100	100	100	100	100	100	2.6
5.5	82	97	99	100	100	100	100	100	100	100	5.8
6.0	54	79	90	95	98	99	100	100	100	100	13.0
6.5	29	50	65	75	82	87	91	94	96	97	29.0
7.0	14	27	37	46	54	60	66	71	75	79	64.7
7.5	7	13	19	24	29	34	38	43	46	50	144.3
8.0	3	6	9	12	14	17	20	22	24	27	322.2

According to the results, the probability of medium-sized (*M*_*s*_ ≥ 5.0) earthquakes within 10 years is 98%, and the probability of strong (*M*_*s*_ ≥ 6.0) earthquakes within 10 years is 54% for the region in general. In addition, the probability of a major earthquake (*M*_*s*_ ≥ 7.0) was calculated as 14% for 10 years and 54% for 50 years. The recurrence periods for moderate (*M*_*s*_ ≥ 5.0) and strong (*M*_*s*_ ≥ 6.0) earthquakes were determined as 2.6 to 13 years, and 74.6 years for a large earthquake (*M*_*s*_ ≥ 7.0).

For the two major earthquakes (Erzincan, *M*_*s*_ = 7.9 and Tokat, *M*_*s*_ = 7.0) in the NAFZ that affected the region, the most and caused significant losses, a deterministic earthquake hazard assessment covering the study area was performed. Within this framework, possible earthquake intensity and acceleration values were calculated. To decrease the soil-independent intensity and horizontal acceleration representing the study areas, the following relations were used:

7 $$I=0.34+1.54\;{M}_{s}-1.24\;{\text{LnD}}\;(\mathrm{Erdik\;et\;al}. 1983)$$

8 $${\text{Log}}\;PA=0.56\;M-0.827 \mathrm{\;Log\;D}-0.236\;(\dot{{\text{I}}}\mathrm{nan\;et\;al}. 1996)$$

where *I* means MSK (Medvedev-Sponheur-Karnig) intensity scale (macroseismic intensity scale), *M*_*s*_ means surface wave magnitude, *D* is the closest (or perpendicular) (km) distance to the fault, and *PA* is the maximum horizontal acceleration (gal = cm/s^2^).

The intensity and acceleration values calculated according to Eqs. (7) and (8) for both considered earthquakes are summarized in Table 4.

**Table 4:** Predicted intensity and acceleration values for each province (1 g = 1000 cm/s^2^)

Province	Erzincan earthquake (*M*_*s*_ = 7.9)		Tokat earthquake (*M*_*s*_ = 7.0)	
	Intensity	Acceleration (g)	Intensity	Acceleration (g)
Samsun	V	0.09–0.15	VII	0.19–0.40
Ordu	VI	0.16–0.21	VI	0.07–0.10
Giresun			V	
Trabzon	VI	0.20–0.30	IV	0.04–0.05
Gümüşhane	VIII	0.21–0.56		
Bayburt				
Artvin	V	0.15–0.16		0.03–0.04

According to the data in Table 4, the locations where the intensity and acceleration are likely to be the highest for the Erzincan earthquake are Bayburt and Gümüşhane (*I* = VIII, PA = 0.21–0.56 g), while the locations where they are likely to be the lowest are Samsun and Artvin (*I* = *V*, PA = 0.09–0.16 g). On the other hand, for the Tokat earthquake, the locations where the intensity and acceleration are likely to be the highest and lowest were Samsun (*I* = VII, PA = 0.19–0.40 g), and Trabzon, Gümüşhane, Bayburt and Artvin (*I* = IV, PA = 0.03–0.05 g), respectively.

### Near surface geophysical methods, data acquisition and processing

The geophysical measurements included seismic refraction tomography (SRT), Multichannel analysis of surface wave (MASW) and electrical resistivity tomography (ERT) measurements. They were preferred and used because of their advantages of rapid data acquisition and low cost and providing the useful physical parameters and images to characterize the subsurface in detail. The surveying profiles were decided after the studies of geographic information systems and multi-criteria decision analyses including geomorphology, vegetation, distance to subsurface-surface water resources (drinking water and streams), odour effects, climatic conditions, agricultural usage, distance to settlement areas and ease of transportation. These methods allow obtaining P-wave, S-wave velocities (*V*_*P*_, *V*_*S*_) and electrical resistivity (*ρ*) parameters with high accuracy, which are necessary for qualified subsurface characterization. For this purpose, 25 SRT, MASW and ERT datasets were acquired from the same profile. For seismic measurements, a 12-channel seismograph, 4.5-Hz vertical geophones (P-geophone) and 9.0-kg sledgehammer (20 × 20 × 5-cm steel plate) were used as seismic source. In each spread, the first receiver offset in the end-on and -off shots was 12 m, and the distance between the receivers was 3 m. For the SRT, five shots (source locations, 0, 19.5, 28.5, 37.5 and 57 m) were performed along the profile. For SRT, the time sampling interval was 0.25 ms, and the recording length was 0.5 s. For the MASW data, the sampling interval was 0.5 ms and the recording length was 1.0 s. In order to improve the signal/noise ratio of the data at each source point, three successive shots were stacked vertically (Table 5).

**Table 5:** Equipment and parameters for field data acquisition in the study

Surveying method	Equipment and parameters
SRT	
	• Device-Seistronix RAS-24, Seistronix, USA
	• 12 geophones with 4.5 Hz (vertical component)
	• Geophone interval: 3.0 m
	• Offset: 12 m for end-on and end-off shots
	• Sampling interval: 0.5 ms
	• Time length: 0.5 s
	• Five shots (two ends and quarters, middle) in a profile
	• Three vertical stacks for each shot
	• 9 kg sledgehammer, 20 × 20x5 cm steel plate
	• Software: Seisimager 2D (Seisimager/2D 2009)
	• Date of data surveying: October 2016-March 2017
MASW	
	• Device: Seistronix RAS-24, Seistronix, USA
	• 12 geophones with 4.5 Hz (vertical component)
	• Geophone interval: 3.0 m
	• Offset: 12 m for end-on and end-off shots
	• Sampling interval: 0.5 ms
	• Time length: 1.0 s
	• Two shots (end-on and end-off) in a profile
	• Three vertical stacks for each shot
	• 9 kg sledgehammer, 20 × 20 × 5-cm steel plate
	• Software-Seisimager SW (Seisimager/SW 2022)
	• Date of data surveying: October 2016–March 2017
ERT	
	• Device: Ambrogeo-Mangusta, Ambrogeo Instruments, ITALY
	• 23 steel electrodes
	• Wenner-Schlumberger
	• Electrode interval: 3.0, 4.0, 5.0 m
	• Software: Res2DINV (Loke 1997)
	• Date of data surveying: October 2016–March 2017

**Fig. 6:**
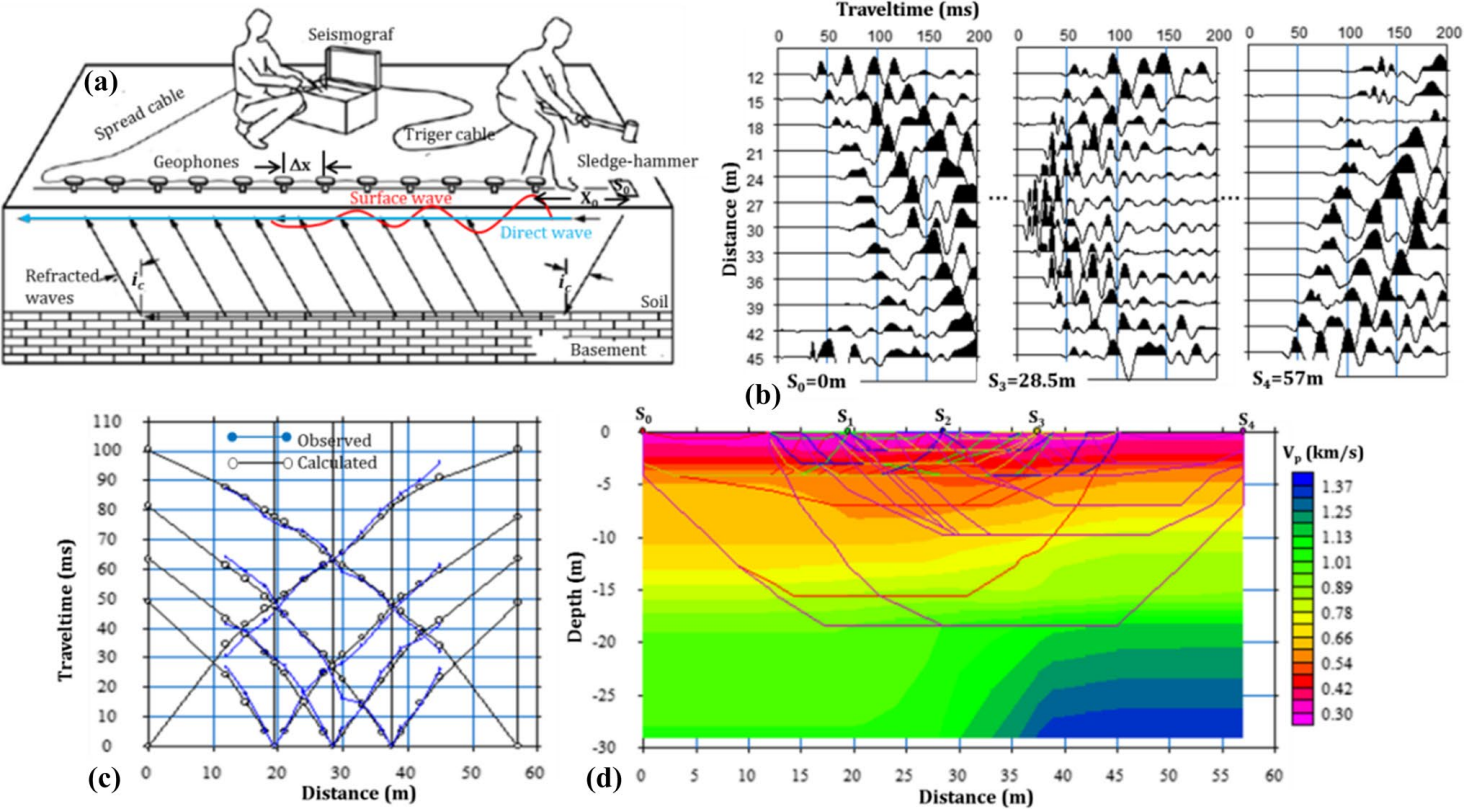
Methodology of the SRT method. **a** Data acquisition layout in the field. **b** The data according to source position (shots 3 and 4 are not shown and the time axis is truncated at 200 ms because of displaying purposes). **c** Observed and calculated (by inversion) first arrival times. **d** 2D SRT section shows variation of the P-wave velocity in depth and spatial dimension (the rays on the section indicate what depth has been reached)

SRT provided a 2D P-wave velocity models of the subsurface in the sites, which are used to detect the bedrock depth and topography, geological strata and boundaries, fracturing and/or weathering, holes and/or caves, groundwater table, consolidated-unconsolidated zones, excavation-rippability classification and dynamic-elastic parameters of soil and rocks (P- and S-wave velocities, *V*_*P*_/*V*_*S*_ ratio, Poisson ratio, shear, Young’s, Bulk modulus, Lamé constant, etc.) (Deidda and Ranieri 2005; Lanz et al. 1998; Olona et al. 2010; Babacan et al. 2018; Kondracka et al. 2021) by interpreting the variations of the P-wave velocity. The methodology of the SRT is explained in Fig. 6 the refraction data of profile 1 in Bafra site. Briefly, the seismic data in Fig. 6b were acquisited, then first arrival times of the critically refracted waves (black line in Fig. 6a) and direct waves (blue line in Fig. 6a) are manually or automatically picked from each seismic data, and distance-time graph is drawn as in Fig. 6c. Lastly, 2D P-wave velocity sections (Fig. 6d) are obtained by tomographic inversion using the nonlinear least square algorithm. In Fig. 6d, the blue coloured area in the lower right corner of the SRT section does not actually show a high velocity compared to the overlying units, and this has been taken into account in the interpretation of the sections. Furthermore, the absence of critical refracted in some layers relative to the ray paths is entirely due to the lack of sufficient velocity difference.

**Fig. 7:**
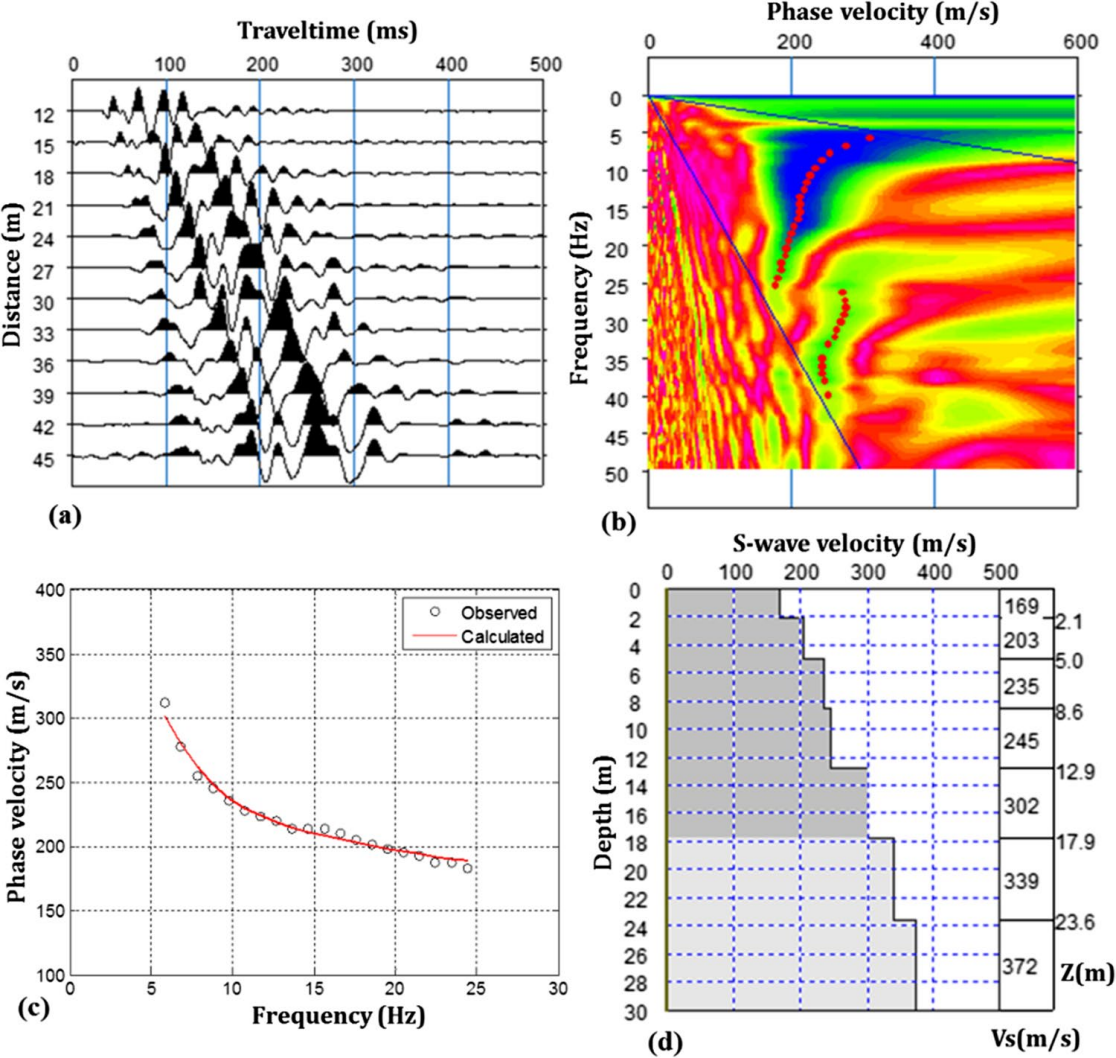
Steps for MASW method. **a** Data acquisition: shot record from the field. **b** data processing: *f*-*v* imaging by phase-shift transformation technique. **c** observed (or selected) and calculated (or inverted) fundamental mode curve. **d** 1D V_S_-depth profile. This profile may be expressed as a shear wave sounding and is generally placed in the middle of the geophone spreading

MASW method, which consists of the data acquisition, dispersion analysis and inversion steps, was used to estimate the depth profile of shear wave velocity (1D Vs) in order to determine the soil thickness, soil-rock definition, geotechnical properties (i.e. softness-stiffness) of soil and rocks in a site (Socco and Strobbia 2004; Zhan et al. 2008; Foti et al. 2011; Dumont et al. 2018). The most important advantage of this method over others methods is that the near-surface resolution (top 10 m) is much improved (Kondracka et al. 2021) and is not affected by velocity reversal problem as in seismic refraction (Glangeaud et al. 1999; Park et al. 1999). In MASW, the dispersion of surface wave field (Rayleigh type surface wave in this study) is concerned and generally the recording time, and sampling time can be chosen to be longer than the seismic refraction. The dispersion of the surface waves is extracted from the data presented in Fig. 7a by applying the frequency-velocity (f-v) transform (phase-shift technique in this study) (Fig. 7b), and the velocity value corresponding to the maximum energy at each frequency is selected as the phase velocity, named as fundamental mode dispersion curve (Fig. 7c). The 1D Vs profile in Fig. 7d also is obtained by inversion of the fundamental mode dispersion curve.

This resulting V_S_-depth profile is assigned to the midpoint of the receiver array and is used to calculate *V*_*S*30_ (average S-wave velocity for 30 m depth) given by Eq. (9) (Borcherdt 1994) which is necessary for soil classification of a site. In this study, soil class assessment for potential landfill areas was accomplished according to TBEC (Türkiye Building Earthquake Code) (2018) adapted from NEHRP (National Earthquake Hazard Reduction Program) (BSSC 2003) (Table 6). According to the new classification of NEHRP (BSSC 2020), B, C and D soil classes are divided into subclasses such as BC, CD and DE. However, TBEC (2018) fully matches the old classification of NEHRP, but Table 6 is arranged in this study, taking into account the new classification of NEHRP. The expression letter “*Z*” in TBEC is the Turkish equivalent of “Soil” in English and is used as a prefix. Thus, by using *V*_*S*30_, the parameters (amplification, dominant vibration frequency or period values) necessary to characterize the behaviour of soils in the potential landfill area under dynamic loads can be easily calculated using experimental formulas commonly used in the literature:

**Table 6:** Site soil classification codes and definitions according to the *V*_*S*30_ value were adapted from TBEC (2018) and NEHRP (BSSC 2020). In the table, the soils include sand, clay and gravel units. The definition of the lithology is attributed to the mechanical properties, and borehole logs are not used

NEHRP (BSSC 2020)			TBEC (2018)		
Site class	Definition	*V*_*S*30_ (m/s)	Site class	Definition	*V*_*S*30_ (m/s)
A	Hard rock	≥ 1524	ZA	Hard rock	≥ 1500
B	Medium hard rock	914 ≤ *V*_*S*30_ < 1524	ZB	Less weathered or medium hard rock	760 ≤ *V*_*S*30_ < 1500
BC	Soft rock	640 ≤ *V*_*S*30_ < 914			
C	Very stiff soils	442 ≤ *V*_*S*30_ < 640	ZC	Very stiff soils or very fractured weak rocks	360 ≤ *V*_*S*30_ < 760
CD	Stiff soils	305 ≤ *V*_*S*30_ < 442			
D	Medium stiff soils	213 ≤ *V*_*S*30_ < 305	ZD	Medium stiff or stiff soils	180 ≤ *V*_*S*30_ < 360
DE	Soft soils	152 ≤ *V*_*S*30_ < 213			
E	Very soft soils	< 152	ZE	Very soft soils	< 180
F	Soils requiring site response analysis	< 152	ZF	Soils requiring site response analysis	

For site class code *E* or *ZE*: Soil or any profile with more than 3 m of soft clay is defined as soil with PI > 20, *w* ≥ 40% and *c*_*u*_ < 25 kPa. For site class code *F* or *ZF*: soils with the risk of collapse and potential collapse under the influence of earthquakes (liquefiable soils, highly sensitive clays, collapsible weak cemented soils, etc.), clays with a total thickness of more than 3 m of peat and/or high organic content, high plasticity (PI > 50) clays with a total thickness of more than 8 m, very thick (> 35 m) soft or medium solid clays

*PI* Plasticity Index, *w* water content, *c*_*u*_ undrained shear strength

9 $${V}_{s30}=\frac{\sum\limits_{i=1}^{N}{h}_{i}}{\sum\limits_{i=1}^{N}\frac{{h}_{i}}{{V}_{si}}}=\frac{30}{\sum\limits_{i=1}^{N}\frac{{h}_{i}}{{V}_{si}}}$$

In the equation, *h*_*i*_ and *V*_*si*_ are respectively layer thickness and shear wave velocity for each layer. *N* indicates the number of layers up to 30 m deep.

**Fig. 8:**
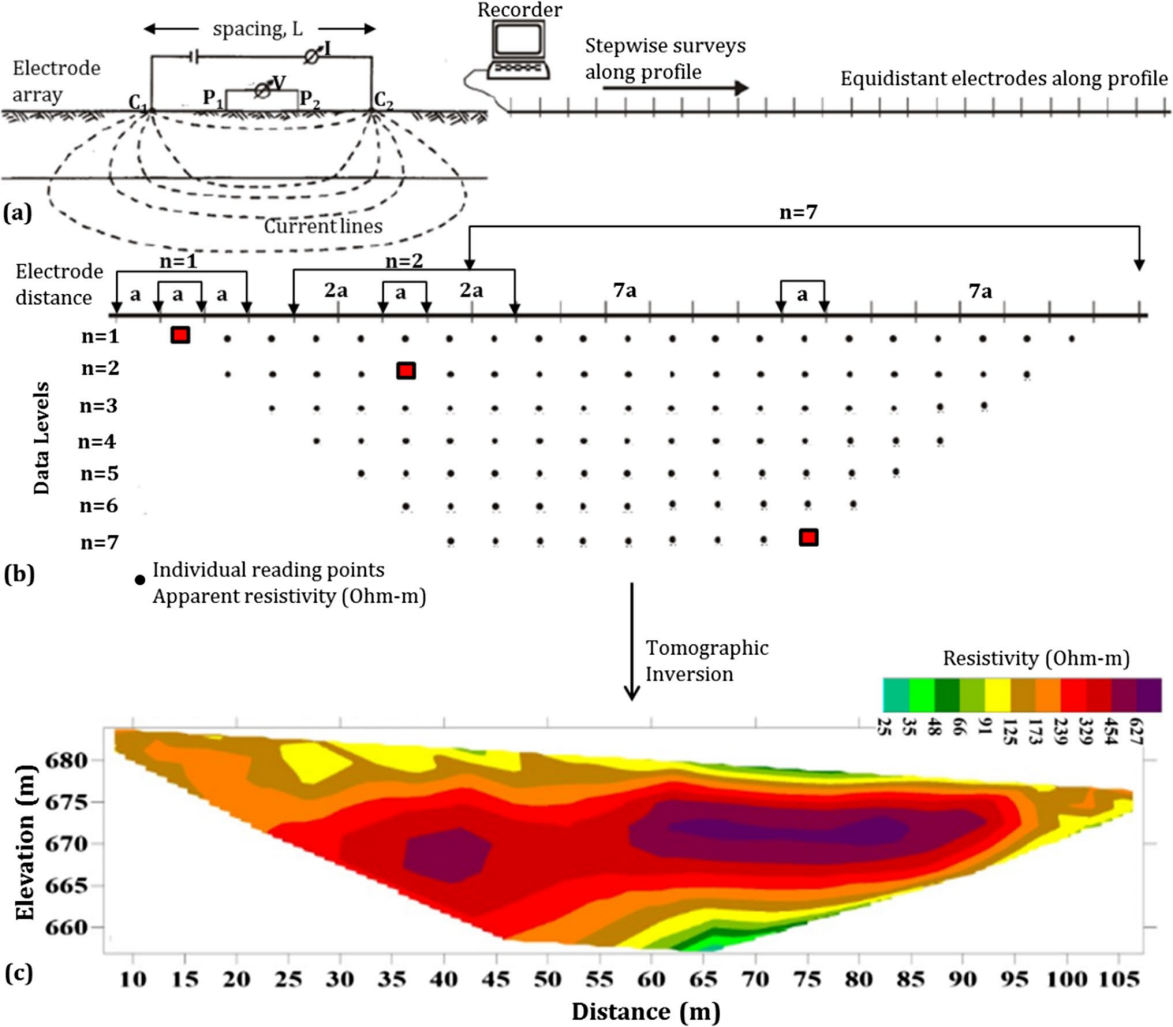
Multi-electrode ERT data acquisition scheme. **a** Current (I; C_1_-C_2_)-potential electrodes (V; P_1_-P_2_), connections and current flow lines. **b** Distribution of apparent resistivity data points read from the subsurface by Wenner-Schlumberger electrode array. **c** 2D ERT section from tomographic inversion. The black dots in **b** represent the depth and spatial position corresponding to the apparent resistivity value obtained from each measurement result. The red squares are a representative display of the position corresponding to the data read in relation to the increase (or n level) in the current electrode spacing

Electrical resistivity tomography method is a geophysical method considering the variation of the electrical resistivity of subsurface materials with the widest use in subsurface explorations and solution of geotechnical problems such as characterizing the subsurface structure of landfills (Vargemezis et al. 2015); detecting groundwater, clay content and seepage deposits (Campbell et al. 1999; Kaya et al. 2007); examining the potential sites for solid waste disposal facilities (Smith and Randazzo 2003; Bichet et al. 2016; El-Kelani and Khader 2019; Hu et al. 2019) and evaluating the locations of regions with different moisture levels in landfills, fractured-cracked structure, permeable-impermeable, mapping resistant and weak lithological units (Guerin et al. 2004; Loke 2004; Olona et al. 2010; Hossain et al. 2011). Therefore, in this study, ERT method was used specially to map the underground water and clay content and weathering degree of the rocks. The methodology of the ERT data acquisition and evaluation systematic is demonstrated in Fig. 8. Shortly, Fig. 8a shows the current and potential electrode positions (Wenner-Schlumberger in this study) within the multi-electrode systems which provide fast and less laborious (Loke and Barker 1996; Loke 1997) data acquisition. Figure 8 b shows the distribution of data points corresponding to the depth levels penetrated for different electrode intervals. To account for topographic changes over wide spans, the elevation values of each electrode are written to the data file. Obtained apparent resistivity values are converted to real resistivity values of the ground by tomographic inversion technique. By applying this process in 2D, ERT sections are created, and they are now ready for interpretation (Fig. 8c). When the resistivity changes are evaluated together with additional information (e.g. seismic velocity), it has the potential to make a significant contribution to the general description of both the physical and lithological nature of the geological material (soil: sand, clay, silt, gravel and rock: volcanic, sedimentary).

The ERT data were acquisited with Wenner-Schlumberger electrode array using 23 electrodes of the Ambrogeo trademark, Mangusta model 48-electrode device. This array was used because it is highly sensitive to both horizontal and vertical structural-lithological changes and has a wider horizontal coverage and signal strength than other arrays. The electrode spacings were 3.0 m (Işıktepe-1, 2 in Ordu; Ağalık-1, 2 in Giresun; Ovacık-1, 2 in Trabzon), 4.0 m (Kazantaş-2 in Gümüşhane) and 5.0 m (Vezirköprü-1,2 and Bafra-1,2 in Samsun; Esence-1,2 in Ordu; Çamburnu-1,2 in Trabzon; Murgul-1,2 in Artvin; Yenice-1,2 and Kazantaş-1 in Gümüşhane; Center-1,2 and Balkaynak-1 in Bayburt), depending on the terrain conditions at the measurement sites.

## Results

**Fig. 9:**
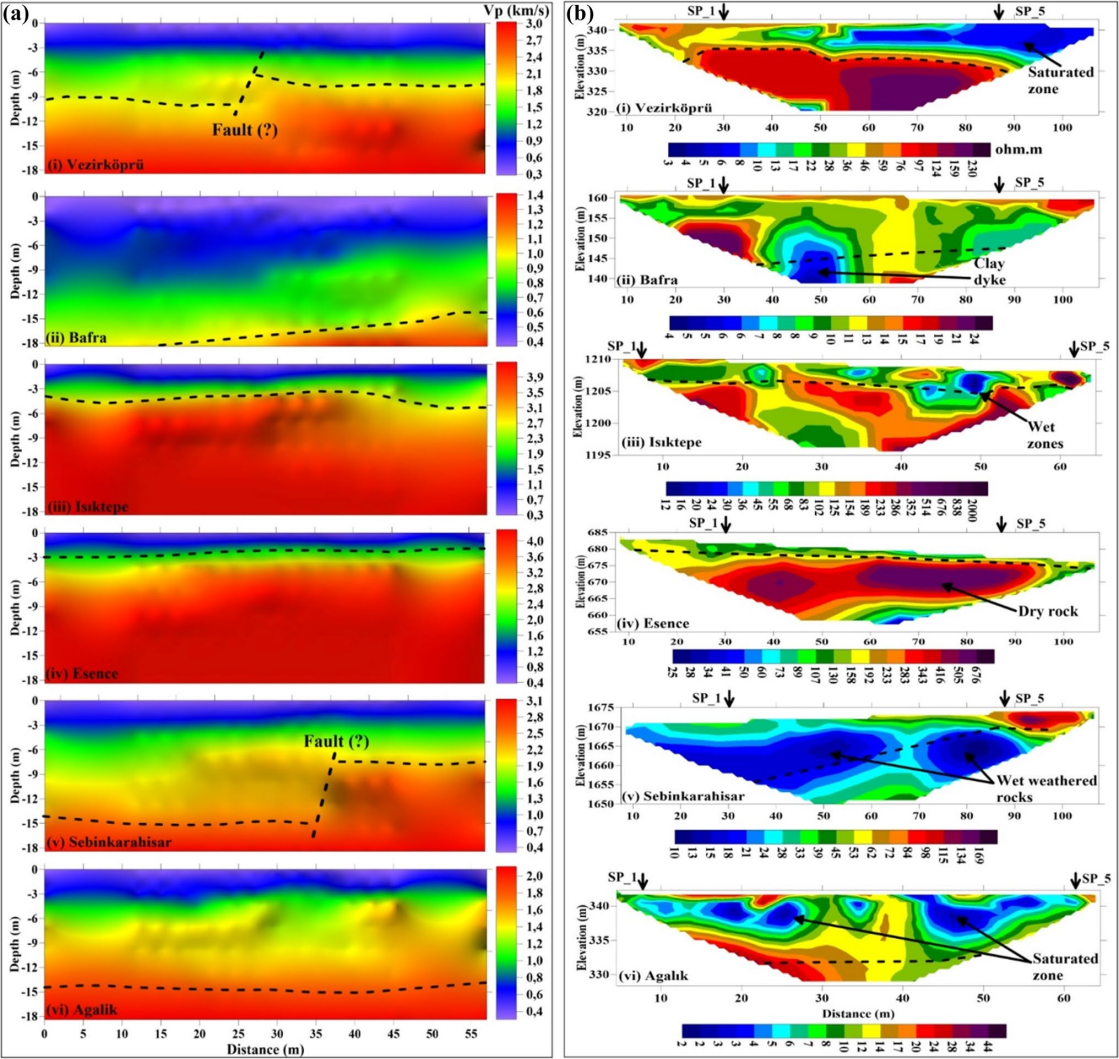
Sections of selected profiles from each survey location. **a** SRT and **b** ERT. The bedrock level (dotted line-dotted line) on the SRT sections is also indicated in the ERT sections, considering the approximately equivalent depth level. Saturated, moist and dry zones are indicated according to the resistivity changes. In addition, probable fault indication was marked in the SRT sections. SP_1 and SP_5 points on the ERT sections indicate the locations of the first and last shots of the five shots along the profiles

**Fig. 10:**
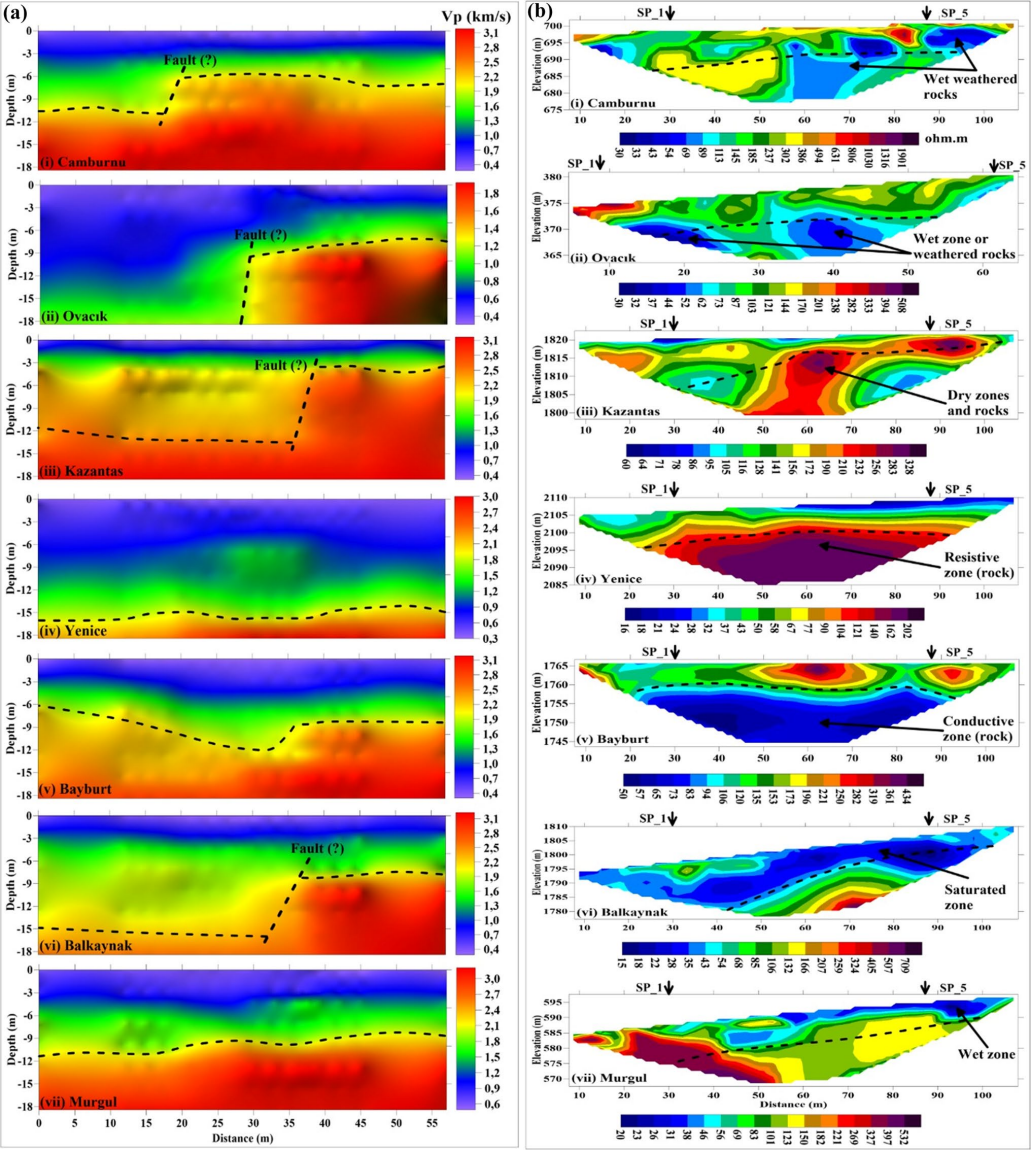
Sections of selected profiles from each survey location. **a** SRT and **b** ERT. The bedrock level (dotted line-dotted line) on the SRT sections is also indicated on the ERT sections, considering the approximately equivalent depth level. According to the resistivity changes, saturated, moist and dry zones are indicated. In addition, probable fault indication was marked on the SRT sections. SP_1 and SP_5 points on the ERT sections indicate the locations of the first and last shots of the five shots along the profiles

Within the scope of this study, quantitative and qualitative analyses of the seismic and electrical resistivity data acquisited in the seven provinces in 25 profiles were performed, and 13 of the obtained cross sections are presented in Figs. 9 and 10. While obtaining SRT, ERT sections and 1D-V_S_ profiles, the RMS (root mean square) error amounts as a result of inversion were less than 3%, 7% and 5%, respectively. According to the *V*_*S*30_ value for all sites, the classification, dominant vibration period (*T*_0_) and amplification (*A*_0_) of the soil and physical description of the soil are presented in Table 7. Also, in Table 7, *Ta* and *Tb* values calculated by using *T*_0_ indicate the corner frequencies required for the construction static project. According to these frequency values, ground-structure resonance period ranges are determined. In addition, the interpretations of the obtained SRT and ERT cross sections are presented in Table 8. It can be said that the results obtained from SRT and ERT are generally compatible. As seen in ERT sections, the range of resistivity values is quite wide (~ 2–2000 Ohm-m). Therefore, in the presentation of the ERT sections, it used individual colour scale to clearly visualize the anomalies. On the other hand, field observations and regional geology were taken into account while interpreting the ERT and SRT sections.

**Table 7:** Soil classifications according to the *V*_*S*30_ value and calculated soil parameters. Although the distance between profiles was variable, it was less than 100 m

Province	Survey location	Profile no	*V*_*S*30_ (m/s)	Soil Amplification *A*_0_ = 68*V_*S*30_^−0.6^ (Midorikawa 1987)	Soil period *T*_0_ = 4H/*V*_*S*30_ (s) (*H* = 30 m)	*T*_*a*_ − *T*_*b*_ (s) *T*_*a,b*_ = (0.5–1.5)*T_0_	Soil class by NEHRP and TBEC	Definition
Samsun	Vezirköprü	1	551	1.54	0.21	0.10–0.32	C, ZC	Very stiff soils/very fractured weak rocks
		2	377	1.93	0.31	0.15–0.47		
	Bafra	1	251	2.47	0.47	0.23–0.71	D, ZD	Medium stiff/stiff soil
		2	223	2.65	0.53	0.26–0.80		
Ordu	Işıktepe	1	1327	0.90	0.09	0.04–0.13	B, ZB	Medium hard rock/less weathered
	Esence	1	572	1.50	0.21	0.10–0.31	C, ZC	Very stiff soils/very fractured weak rocks
		2	570	1.51	0.21	0.10–0.31		
Giresun	Şebinkarahisar	1	396	1.87	0.30	0.15–0.45	CD, ZC	Stiff/very stiff soils
		2	857	1.18	0.14	0.07–0.21	BC, ZB	Soft rock/less weathered rock
	Ağalık	1	274	2.34	0.43	0.21–0.65	D, ZD	Medium stiff/stiff soil
		2	300	2.21	0.40	0.20–0.60		
		3	435	1.77	0.27	0.13–0.41	CD, ZC	Stiff/very stiff soils
Trabzon	Çamburnu	1	484	1.66	0.24	0.12–0.37	C, ZC	Very stiff soils/very fractured weak rocks
		2	1016	1.06	0.11	0.05–0.17	B, ZB	Medium hard rock/less weathered
	Ovacık	1	384	1.91	0.31	0.15–0.46	CD, ZC	Stiff/very stiff soils
		2	483	1.66	0.24	0.12–0.37	C, ZC	Very stiff soils/very fractured weak rocks
Gümüşhane	Kazantaş	1	700	1.33	0.17	0.08–0.25	BC, ZC	Soft rock/very fractured weak rocks
		2	761	1.27	0.15	0.07–0.23	BC, ZB	Soft rock/less weathered rock
	Yenice	1	422	1.80	0.28	0.14–0.42	CD, ZC	Stiff/very stiff soils
		2	417	1.82	0.28	0.14–0.43		
Bayburt	Merkez	1	614	1.44	0.19	0.09–0.29	C, ZC	Very stiff soils/very fractured weak rocks
		2	805	1.22	0.14	0.07–0.22	BC, ZB	Soft rock/less weathered rock
	Balkaynak	1	371	1.95	0.32	0.16–0.48	CD, ZC	Stiff/very stiff soils
Artvin	Murgul	1	852	1.18	0.14	0.07–0.21	BC, ZB	Soft rock/less weathered rock
		2	844	1.19	0.14	0.07–0.21

**Table 8:** Interpretation of SRT and ERT sections in Figs. 9 and 10 and evaluation of suitability in terms of the waste landfill site

Province	Location	H(m)	V_P_ (km/s)	Description for SRT	*ρ* (Ohm-m)	Description for ERT	Joint interpretation	Suitability for landfill
Samsun	Vezirköprü	0.0–3.0	0.3–1.2	Soft to stiff soils	3–10	Low resistive soils	According to the resistivity change, soft and compact soil units are mostly dry and partially moist at distances of 10–55 m, whereas the zone between 55 and 105 m distance and 9.0 m depth from the surface is saturated. The fact that *V*_*P*_ = 1.4–1.6 km/s and *ρ* = 3–6 Ohm-m in this range indicates that the geological material is clay-rich. A clear discontinuity (probably a fault) is observed in the SRT section at 30–35 m. After 9 m depth, there are rock units suitable for foundation. Although the environment is considered suitable as a landfill area in terms of seismic parameters, resistivity data indicate the presence of groundwater up to 4.5 m deep in the environment	Partly suitable after geotechnical reclamation
		3.0–9.0	1.2–1.8	Very stiff soils or highly weathered rock	10–60	Moderately resistive soils		
		> 9.0	> 1.8	Moderately weathered rock	> 60	Resistive rock		
	Bafra	0.0–2.0	0.4–0.7	Very soft soils	4–7	Very low resistive soils	The maximum–minimum resistivity variation was approximately 4–24 Ohm-m. According to the SRT section, the ground was very soft from the surface to a depth of 12 m (no bedrock!). According to ERT data, this low resistivity implies that the medium is highly saturated. This site is very risky in terms of ground settlement, collapse, fracture and liquefaction in case of both vertical and horizontal loads during the passage of future earthquake waves	Not suitable
		2.0–12	0.7–1.0	Soft soil	7–18	Low resistive soils		
		> 12	> 1.0	Very stiff soils				
Ordu	Işıktepe	0.0–1.5	0.8–1.4	Very stiff soils	10–45	Moderately resistive soils	According to the seismic parameters, the environment has a very solid soil near its surface. Especially after 3.2 m depth, the P-wave velocity implies a hard rock structure. The ERT section show local moistening at near the surface, but these were considered to be seasonal. Therefore, it is believed that no groundwater exists in the environment	Suitable
		1.5–3.2	1.4–2.2	Moderately weathered rock to rock	45–180	Resistive rock		
		> 3.2	> 2.2	Hard rock	> 180	Highly resistive rock		
	Esence	0.0–1.5	0.8–1.6	Very stiff soils	66–173	Resistive soils	There is massive bedrock starting at a depth of about 3.0 m. Moreover, the resistivity data show that the medium is dry	Suitable
		1.5–3.5	1.6–2.5	Rock to hard rock	> 173	Highly resistive rock		
		> 3.5	> 2.4	Very hard rock				
Giresun	Şebinkarahisar	0.0–1.5	0.4–1.2	Soft-stiff soils	10–25	Low resistive soils	Medium-stiff soil and weathered rock were observed from the surface to a depth of 7 m. A discontinuity zone (probably lithological or facies change) was observed at intervals of 25–30 m in the SRT section and 65–70 m in the ERT section. However, the low resistivity zones (< 25 Ohm-m) within the first 10 m are thought to be caused by surface seepage water which moistens the weathered rock	Partly suitable after geotechnical reclamation
		1.5–7.0	1.2–2.1	Stiff soils to moderately weathered rock	25–70	Moderately resistive rock		
					> 70	Resistive rock		
		> 7.0	> 2.1	Hard rock				
	Ağalık	0.0–4.5	0.4–0.9	Soft-stiff soils	2–5	Very low resistive soils	Units with a resistivity of *ρ* ≤ 20 Ohm-m observed from the surface to a depth of 13 m indicate the presence of clay-containing, water-saturated units. The low resistivity of the deeper rock material is associated with its highly weathered structure and therefore the presence of water seeping from its cracks. The risk of the site for any construction is high	Not suitable
		4.5–13.0	0.9–1.6	Very stiff soils	10–20	Low resistive soils		
		> 13.0	> 1.6	Highly weathered rock	> 20	Resistive soils		
Trabzon	Çamburnu	0.0–3.0	0.5–1.2	Soft to stiff soils	30–70	Moderately resistive rock	The surface units are stiff and dry. The units below show very stiff and weathered rock characteristics. In addition, the decrease in the resistivity values of the rocks, which appear at high velocity from the surface to the depth, especially at distances beyond 55 m, was considered to be caused by water leaking from the surface	Suitable
		3.0–10.0	1.2–2.2	Moderately weathered rock	70–120	Resistive soils		
		> 10.0	> 2.2	Hard rock	> 120	Highly resistive rock		
	Ovacık	0.0–5.0	0.4–0.7	Very soft soils	30–70	Moderately resistive soils	There are mostly thick and dry soil units up to a depth of 5 m in the environment. In the SRT section (Fig. 10a(ii)), a fault, possibly a normal fault, was observed in the 30–35 m range, causing lateral lithological change. In addition, the 1.4–1.8 km/s velocity unit placed at ~ 9 m from the surface after 30 m distance is seen as low resistivity (30–70 Ohm-m) in the ERT section (Fig. 10b(ii)). This was clearly caused by the infiltration of seasonal water from the surface into soil materials or weathered rocks	Not suitable
		5.0–15.0	0.7–1.2	Soft to stiff soils	70–150	Resistive soils		
		> 15.0	> 1.2	Very stiff soils to highly weathered rock	> 150	Highly resistive soils		
Gümüşhane	Kazantaş	0.0–1.5	0.5–1.3	Soft to stiff soils	60–105	Moderately resistive soils	From the surface to a depth of 3.5 m, medium-stiff-very stiff dry soils and weathered rocks were observed, while deeper (> 3.5 m) dry and rather hard rocks were observed	Suitable
		1.5–3.5	1.3–2.2	Very stiff soils to moderately weathered rock	105–170	Resistive soils		
		> 3.5	> 2.2	Hard rock	> 170	Highly resistive rock		
	Yenice	0.0–5.0	0.3–0.9	Soft soils	15–35	Low resistive soils	The environment was very evenly bedded, and a medium-stiff-very stiff soil structure was observed from the surface to a depth of ~ 15 m. After this depth, moderately weathered dry rock units were placed. According to the ERT section, resistivity levels of 10–35 Ohm-m indicate moistened rocks	Suitable after geotechnical reclamation
		5.0–15.0	0.9–1.9	Stiff to very stiff soils	35–105	Moderately resistive soils		
		> 15.0	> 1.9	Moderately weathered rock to hard rock	> 105	Resistive rock		
Bayburt	Merkez	0.0–3.5	0.4–1.2	Soft to stiff soils	50–105	Moderately resistive soils	According to the SRT and ERT sections, the environment showed very dry soil characteristics up to a depth of 8 m. After this depth, the materials show rock structure and have massive character. However, the low resistivity of the massive rock unit at a depth of more than 8 m was associated with the fact that the rock contained conductive minerals rather than water, considering where the location of this site is an old mine (with sulfide mineral content)	Suitable after testing the mineral content of the rock
		3.5–8.0	1.2–2.1	Very stiff soils to moderately weathered rock	105–195	Resistive soils		
		> 8.0	> 2.1	Rock to hard rock	> 195	Highly resistive rock		
	Balkaynak	0.0–3.0	0.4–1.2	Soft to stiff soils	15–55	Low resistive soils	Especially, in the 3–9-m depth range, the average P wave velocity of 1500 m/s and the low resistivity value (5–13 Ohm) indicate the presence of water-saturated soil material here. In addition, a lateral discontinuity indication was observed in the 30–35-m distance range in the SRT section. Although the bedrock in this area is hard, geotechnical problems are likely to occur up to 9-m depth. Moreover, observed in the ERT section, the slope of both the surface and bedrock topography indicate a possible landslide hazard	Suitable after geotechnical reclamation
		3.0–9.0	1.2–2.0	Very stiff soils to moderately weathered rock	55–105	Moderately resistive soils		
		> 9.0	> 2.0	Rock to hard rock	> 105	Resistive rock		
Artvin	Murgul	0.0–5.0	0.6–1.4	Stiff to very stiff soils	20–55	Moderately resistive soils	From the surface to a depth of 5 m, there are units with high moisture content, and deeper, resistant, moderately weathered and massive rock units are located. In addition, the slope of both the surface and bedrock topography and the water content of the materials on the bedrock indicate the presence of landslide hazards	Suitable after geotechnical reclamation
		5.0–12.0	1.4–2.2	Moderately weathered rock	55–150	Resistive soils		
		> 12.0	> 2.2	Rock to hard rock	> 150	Highly resistive rock		

Geotechnical reclamation may require operations that include groundwater drainage, soil improvement (compaction, jet grouting, injection, pilling, etc.) and removal of soft top soil cover

The subsurface geometry was defined as a three layers according to the P-wave velocity variation in the SRT sections. In particular, the bedrock depth and topographic changes were indicated in the sections, and the mechanical properties of the soil and rock layers (softness-stiffness of the soils and weathering degree of the rocks) were evaluated according to the P-wave velocity change utilized by the table of the seismic velocity-rippability-geotechnical definition given by Karslı et al. (2021).

## Discussion

In order to determine the suitable solid waste landfill areas, seismological and near-surface geophysical measurements were carried out in alternative areas determined by preliminary studies in different provinces of the Eastern Black Sea Region. Since this study was carried out with the aim of making the suitable site selection of the waste landfill areas, it differs from the studies on the determination and solution of the problems which arise in the existing or abandoned landfills. These differences are not related to the geophysical methods used and the processing of the obtained data, but to the interpretation of the findings obtained from the data in order to prevent problems that may arise after landfill.

The statistical and deterministic evaluations (earthquake hazard analysis) based on seismological data were made by considering the geographical boundaries of the provinces where the landfill areas foreseen for solid waste are located. Therefore, rather than the locations of the landfill areas (or surveying locations), the distance of the geographical boundaries of the provinces to the earthquake epicentre (especially for NAFZ) was effective in these assessments. It should be noted that, although all other provinces (Samsun, Ordu, Giresun and Artvin) have a coast to the Black Sea, except for Gümüşhane and Bayburt provinces (Trabzon Province is bordered by Gümüşhane and Bayburt Provinces from the south), the southern parts of these provinces are quite close to the NAFZ. On the other hand, the foreseen landfill areas are mostly located in the northern parts of the provinces, in areas closer to the city centres. Therefore, according to Table 2, the small *b*-values characterize the municipalities of Ordu (*b* = 0.47), Gümüşhane (*b* = 0.48) and Trabzon (*b* = 0.48), and the large *b*-values are characteristic of the provinces of Artvin (*b* = 0.87), Bayburt (*b* = 0.68), Samsun (*b* = 0.65), Giresun (*b* = 0.57) respectively. The fact that the *b*-values calculated for Ordu, Gümüşhane and Trabzon are normally smaller than for the other provinces, especially those close to the NAFZ, is related to the limited number of earthquakes occurring within the municipal borders and near around of Ordu, Gümüşhane and Trabzon, especially with *M*_*s*_ ≥ 6.0. Therefore, it may be misleading to make a comparative qualitative seismicity assessment of the *b*-values calculated for each province. Taking into account the values of parameter “*b*” determined locally, it is clear that the frequency of earthquakes causing the damage in the study areas is low and in good agreement with the probability calculations presented in Table 3. On the other hand, considering the probability of earthquake occurrence in 10-year period values in Fig. 5 and the *b*-value in Eq. (6), it is understood that the seismic hazard of the region is high, especially for a medium-sized earthquake in the near future. Furthermore, when evaluated independently of the soil, it is clear that the intensity and acceleration values in Table 4 were high in locations close to the earthquake epicentre and diminished as they moved away from it. However, the intensity and acceleration values may increase in local soils, far from the earthquake epicentre, which may cause the potential for soil amplification, increasing the vibration period and liquefaction and focusing due to the basement (or bedrock) topography. This situation requires site characterization and is explained by the nonlinear behaviour of the soils.

From the near-surface geophysical measurements, seismic measurements contributed to the soil characterization of the landfill areas, while electrical measurements provided information about water content, lithological changes and degree of rock weathering. In fact, this information directly explains the structure and behaviour of the subsurface against possible loads, in both vertical and horizontal direction. Accordingly, near-surface geophysical measurements performed in the site selection of solid waste landfill areas, compared with other determination methods (city planning, geological, hydrogeological, environmental science, social and health sciences and geographic information systems, etc.), have important advantages in terms of taking geotechnical measures for both the construction design of the landfill areas and the precaution of structural problems which may arise later.

Therefore, according to results shown in Table 7, the lowest *V*_*S*30_ value was measured in Bafra (*V*_*S*30_ = 223 m/s) and the highest in Işıktepe (*V*_*S*30_ = 1327 m/s). Although soil classes varied between D and B, it was mostly observed CD, C and BC soil classes for NEHRP (BSSC 2020) and ZC and ZB soil classes for TBEC (2018). These soil classes indicate the existence of geological environments containing moderately stiff to very stiff soil layers, which are not thick and cover less weathered rocks at the basement, which is consistent with the general geology of the study areas. The soft soils (D or ZD) are in Bafra and Ağalık, while the hard or medium hard rocks (B or ZB) are in Işıktepe and Çamburnu. Some soils (Şebinkarahisar-2, Kazantaş-2, Bayburt Merkez-2, Murgul) are ZB (less weathered or medium hard rocks) according to TBEC (2018) in the subclass BC (soft rocks) according to NEHRP (BSSC 2020). In such soils, it will be more appropriate to perform the necessary calculations considering the BC soil class in designed geotechnical projects in terms of construction safety. On the other hand, the minimum–maximum of them was calculated as *T*_0_ = 0.09–0.47 s and *A*_0_ = 1.06–2.47, respectively. The soil amplification values were calculated according to the Midorikawa (1987) approach, and the lower and upper limits of the soil dominant vibration period value (*T*_*a*_ = 0.5*T_0_, *T*_*b*_ = 1.5*T_0_), design period and frequency values, were obtained according to the quarter wavelength approach of Keçeli and Cevher (2018). For all profiles, the minimum and maximum *T*_0_ were for Işıktepe (*T*_0_ = 0.09 s) and Bafra (*T*_0_ = 0.53 s), respectively, while the minimum and maximum *A*_0_ were calculated at Çamburnu (*A*_0_ = 1.06) and Bafra (*A*_0_ = 2.65). *A*_0_ < 1.0 means that there is no amplification in the soil, although numerically, the smallest soil amplification (*A*_0_ = 0.9) in Table 7 is obtained for Işıktepe. As can be seen, the largest values for soil amplification and soil dominant vibration period were calculated at the Bafra location.

From the ERT sections, information was obtained regarding the water content (humidity and saturation) and possible clay content of the geological units according to the resistivity changes. The presence of water-saturated units in the ERT section corresponding to the fault locations determined in the SRT section was remarkable. This is associated with the fact that the faults are important transmission ways that allow the surface waters to infiltrate deep and the aquifer layer to be formed. Thus, the information provided by each geophysical data point in terms of soil and rock characteristics was interpreted to eliminate possible uncertainties. In this context:

1. Zones corresponding to *V*_*P*_ $\widetilde{=}$ 1300–1550 m/s observed in SRT sections and low resistivity (*ρ* < 10 Ohm-m) in ERT sections were considered as saturated with water
2. Zones with low velocity (*V*_*P*_ < 1000 m/s) but high resistivity (*ρ* >  ~ 70 Ohm-m) are considered to be dry soil units
3. Zones with moderate velocity (*V*_*P*_ $\widetilde{=}$ 1200–2100 m/s) and high resistivity are interpreted as very compact soil and soft and/or weathered rocks, which generally do not contain water
4. Zones with high velocity (*V*_*P*_ > 2100 m/s) but low resistivity were evaluated considering the possibility that the rocks contain water or clay in their cracks or pores or conductive minerals
5. The shear wave velocity (*V*_*s*_) is particularly sensitive to the mechanical properties of the soil, but very little sensitive to its water content. However, the *V*_*s*_ value can be lower than 100 m/s, especially in soft clays, while it is more than 1000 m/s in stiff soil and rock materials. Thus, the 1D-*V*_*s*_ depth profile was used to determine the softness-stiffness soils, soil-rock boundaries and soil classification
6. The ERT sections have been very helpful in evaluating the unconsolidated structure of the soil material at the measurement sites, the degree of weathering of the rocks, the presence of clayey intermediation and the water content, but they have not been successful enough to describe the soil-rock interfaces. However, these interfaces were identified from the SRT sections and marked on the ERT sections. On the other hand, the bedrock depth levels were precisely compatible in the SRT and ERT sections, particularly when the rocks were dry

## Conclusions

This study includes seismicity analyses and subsurface characterization using near surface geophysical measurements (SRT, MASW, ERT) for planned waste landfill sites in seven provinces in the Eastern Black Sea Region of Türkiye. Seismicity analyses provided the probability of occurrence of earthquakes with the magnitude of medium, strong and large scale (*M*_*s*_ ≥ 5.0, 6.0, 7.0) which can negatively affect the study areas as well as their recurrence periods. Furthermore, the acceleration and intensity predictions for two scenario earthquakes were performed. These analyses show that the seismic hazard of the region is quite high, especially for a medium-sized earthquake, at a high level for strong earthquakes and relatively low for major earthquakes, according to the 10- and 50-year recurrence probabilities. On the other hand, the calculated soil-independent acceleration and intensity values were quite high in provinces close to the scenario earthquake locations and low in the distant provinces.

While SRT showed soil geometry, bedrock depth and physical properties of geological materials, MASW effectively provided soil classification, soil-rock contrast and their mechanical behaviour (softness-stiffness profile, amplification and vibration dominant period) at 30-m depth. ERT is extremely effective in detecting moist, saturated and dry zones. The combined interpretation of SRT, MASW and ERT allowed to significantly reduce the inadequacy or uncertainty of interpretation possible with only one method. Thus, the subsurface structural condition of the possible landfill areas and the characterization of the soil and rocks were performed accurately and decisively. In particular, while the SRT and ERT results were highly compatible in determining the bedrock depth in dry environments, the ERT results were not successful in the presence of groundwater. According to the geophysical results, four sites (Işıktepe, Esence, Çamburnu and Kazantaş) were found to be suitable, and six sites (Vezirköprü, Şebinkarahisar, Yenice, Bayburt-Center, Balkaynak and Murgul) were found to be suitable after necessary geotechnical reclamation. Three sites (Bafra, Ağalık, Ovacık) were considered to be unsuitable because of the presence of thick, water-saturated soft soil and extremely weathered rocks. Soils at unsuitable sites are risky because they have the potential to amplify earthquake waves and increase the vibration period and are susceptible to possible deformations and landslides after landfilling.

As a result, this study has shown that the seismicity of possible waste landfill sites and the physical and mechanical properties of geological materials at these sites can be investigated in a non-invasive, rapid and easy way using multiple geophysical methods. This information will enable us to minimize and/or prevent possible deformations (fracturing, collapse, settlement, sliding, etc.), environmental problems (such as dirty water leaks) and economic losses after landfilling. Therefore, municipal governments will be able to make more accurate and less risky decisions in making final decisions about waste landfill sites, which are extremely important for the planning and development of a city.

## Data Availability

The data used in this study comprise the geophysical and seismological part of the project and are available from Eastern Black Sea Project Regional Development Administration (DOKAP), but restrictions apply to the availability of these data, which were used under license for the current study, and so are not publicly available.

## References

[CR1] Alptekin Ö (1973) Focal mechanism of earthquakes in Western Turkey and their tectonic implications, Ph.D. Thesis, New Mexico Inst. of Mining and Tech., Soccoro, New Mexico, 190p

[CR2] Ambraseys NN, Jacson JA (1981). Earthquake hazard and vulnerability in the Northeastern Mediterranean: the Corinth earthquake sequence of February-March 1981. Disaster.

[CR3] Aydın Y (2016) Zaman ve magnitüd kestirilebilir model ile Türkiye'de uzun dönem deprem kestirimi, Yüksek Lisans Tezi, Karadeniz Teknik Üniversitesi Fen Bilimleri Enstitüsü Jeofizik Mühendisliği Anabilim Dalı, Trabzon, Türkiye (in Turkish).

[CR4] Ayhan E, Alsan E, Sancaklı N, Üçer SB (1987) Türkiye ve Dolayları Deprem Kataloğu 1881–1980, Boğaziçi Üniversitesi (BÜ) Kandilli Rasathanesi Gök ve Yer Bilimleri Araştırma ve Uygulama Merkezi, İstanbul, Türkiye, 126p (in Turkish)

[CR5] Babacan AE, Gelişli K, Tweeton D (2018). Refraction and amplitude attenuation tomography for bedrock characterization: Trabzon case (Turkey). Eng Geol.

[CR6] Balia R, Littarru B (2010). Geophysical experiments for the pre-reclamation assessment of industrial and municipal waste landfills. J Geophys Eng.

[CR7] Bektaş O, Yılmaz C, Taslı K, Akdağ K, Özgür S (1995). Cretaceous rifting of the Eastern Pontide carbonate platform (NE Turkey): the formation of carbonates breccias and turbidites as evidences of drowned platform. Geologia.

[CR8] Bichet V, Grisey E, Aleya L (2016). Spatial characterization of leachate plume using electrical resistivity tomography in a landfill composed of old and new cells (Belfort, France). Eng Geol.

[CR9] Borcherdt RD (1994). Estimates of site-dependent response spectra for design (methodology and justification). Earthq Spectra.

[CR10] Boudreault J, Dube J, Chouteau M, Winiarski T, Hardy E (2010). Geophysical characterization of contaminated urban fills. Eng Geol.

[CR11] BSSC (Building Seismic Safety Council) (2003) NEHRP recommended provisions for seismic regulations for new buildings and other structures, Part1: Provisions, FEMA 368, Federal Emergency Management Agency, Washington, D.C.

[CR12] BSSC (Building Seismic Safety Council) (2020) NEHRP recommended provisions for new buildings and other structures. Federal emergency management agency (FEMA) of the U.S. Department of homeland security by the building seismic safety council of the national institute of building sciences, Table 20.2–1 in p 124, Washington, D.C.

[CR13] Campbell DL, Horton RJ, Bisdorf RJ, Fey DL, Powers MH, Fitterman DV (1999). Some geophysical methods for tailings/mine waste work. Tailings Mine Waste.

[CR14] Choudhury D, Savoikar P (2009). Equivalent-linear seismic analyses of MSW landfills using DEEPSOIL. Eng Geol.

[CR15] Çınar H, Altundaş S, Ersoy E, Bak K, Bayrak N (2016). Application of two geophysical methods to characterize a former waste disposal site of the Trabzon-Moloz district in Turkey. Environ Earth Sci.

[CR16] Deidda GP, Ranieri G (2005). Seismic tomography imaging of an unstable embankment. Eng Geol.

[CR17] DOKAP (2021) Report of Regional development programme (2021-2023), Eastern black sea project regional development administration (DOKAP), Giresun, Türkiye

[CR18] Dorhofer G, Siebert H (1998). The search for landfill sites—requirements and implementation in Lower Saxony, Germany. Environ Geol.

[CR19] Dorn M, Tantiwanit W (2001). New methods for searching for waste disposal sites in the Chiang Mai-Lamphun basin, Northern Thailand. Environ Geol.

[CR20] Dumont G, Robert T, Mark N, Frédéric N (2018) Assessment of multiple geophysical techniques for the characterization of municipal waste deposit sites. In: Lecture Notes in Civil Engineering. Springer, pp 668–676. 10.1007/978-981-10-6713-6_66

[CR21] El-Kelani R, Khader A (2019) Refraction seismic study over a proposed landfill site in South West Bank. In: Palestine. Springer, Cham, pp 99–101. 10.1007/978-3-030-01656-2_22

[CR22] Emre O, Duman TY, Ozalp S, Elmaci H, Olgun S, Saroglu F (2013) 1/1.250.000 scaled Turkey active fault map. Mineral Research and Exploration General Directorate. http://www.mta.gov.tr/.Accessed 10 Oct 2023

[CR23] Environmental Protection Agency (EPA) (1999) EPA landfill manuals. Landfill restoration and aftercare. EPA, Wexford, Ireland

[CR24] Erdik M, Eren K (1983) Attenuation of intensities for earthquakes associated with the North Anatolian Fault, Middle East Technical University, Earthquake Research Center, Ankara, Türkiye

[CR25] Eyüboğlu Y, Santosh M, Bektaş O, Ayhan S (2011). Arc magmatism as a window to plate kinematics and subduction polarity: example from the eastern Pontides belt, NE Turkey. Geosci Front.

[CR26] Fernández-Baniela F, Arias D, Rubio-Ordóñez A (2021). Seismic refraction and electrical resistivity tomographies for geotechnical site characterization of two water reservoirs (El Hierro, Spain). Near Surf Geophys.

[CR27] Foti S, Parolai S, Albarello D, Picozzi M (2011). Application of surface-wave methods for seismic site characterization. Surv Geophys.

[CR28] Genelle F, Sirieix C, Riss J, Naudet V (2012). Monitoring landfill cover by electrical resistivity tomography on an experimental site. Eng Geol.

[CR29] Glangeaud F, Mari JL, Lacoume JL, Mars J, Nardin M (1999). Dispersive seismic waves in geophysics. Eur J Environ Eng Geophys.

[CR30] Guerin R, Munoz ML, Aran C, Laperrelle C, Hidra M, Drouart E, Grellier S (2004). Leachate recirculation: moisture content assessment by means of a geophysical technique. Waste Manag.

[CR31] Gündoğdu O, Altınok Y (1986) Türkiye ve Çevresi Deprem Veri Seti 1900–1986, İ.Ü. Mühendislik Fakültesi, Jeofizik Müh. Bölümü, İstanbul, Türkiye (in Turkish)

[CR32] Gutenberg B, Richter CF (1954). Seismicity of the earth and related phenomena.

[CR33] Güven İH (1993) Doğu Pontidlerin 1/25 000 ölçekli jeolojisi ve komplikasyonu, MTA, Ankara, Türkiye (in Turkish)

[CR34] Hossain S, Kemler V, Dugger D, Manzur S, Penmethsa K (2011). Monitoring moisture movement within municipal solid waste in enhanced leachate recirculation landfill using resistivity imaging. Sustain Environ Res.

[CR35] Hu J, Wu XW, Ke H, Xu XB, Lan JW, Zhan LT (2019). Application of electrical resistivity tomography to monitor the dewatering of vertical and horizontal wells in municipal solid waste landfills. Eng Geol.

[CR36] İnan E, Çolakoğlu Z, Koç N, Bayülke N, Çoruh E (1996) 1976–1996 Yılları arası ivme kayıtları olan deprem kataloğu. T. C. Bayındırlık ve İskan Bakanlığı Afet İşleri Genel Müdürlüğü Deprem Araştırma Dairesi Başkanlığı, Ankara, Türkiye, 98p (in Turkish)

[CR37] Karslı H, Babacan AB, Şenkaya M, Gelişli K (2021). An evaluation on rippability of geological units by seismic P- and S-wave velocities. Pamukkale Univ J Eng Sci.

[CR38] Kaya MA, Özürlan G, Şengül E (2007). Delineation of soil and groundwater contamination using geophysical methods at a waste disposal site in Çanakkale, Turkey. Environ Monit Assess.

[CR39] Kayabalı K (1996). Engineering geological aspects of replacing a solid waste disposal site with a sanitary landfill. Eng Geol.

[CR40] Keçeli A, Cevher M (2018). Soil dominant period and resonance relation of building height. J Appl Earth Sci.

[CR41] Keskin S, Pedoja K, Bektas O (2010). Coastal uplift along the eastern Black Sea Coast: new marine tarrace data from eastern Pontides (Turkey) and a review. J Coast Res.

[CR42] Ketin İ (1976) San Andreas ve Kuzey Anadolu Fayları Arasında Bir Karşılaştırma, Türkiye Jeoloji Kurumu Bülteni 19: 149-154 (in Turkish)

[CR43] Ketin İ (1977) Genel Jeoloji, İ.T.Ü. Maden Fakültesi Yayını, Cilt 1, Sayı 1096, 597p, İstanbul, Türkiye (in Turkish)

[CR44] Kondracka M, Kleczek IW, Sitek S, Ignatiuk D (2021). Evaluation of geophysical methods for characterizing industrial and municipal waste dumps. Waste Manag.

[CR45] Konstantaki LA, Ghose R, Draganov D, Diaferia G, Heimovaara T (2015). Characterization of a heterogeneous landfill using seismic and electrical resistivity data. Geophysics.

[CR46] Kosugi K, Katsura S, Katsuyama M, Mizuyama T (2006). Water flow processes in weathered granitic bedrock and their effects on runoff generation in a small headwater catchment. Water Resour Res.

[CR47] Kowalczyk S, Cabalski K, Radzikowski M (2017). Application of geophysical methods in the evaluation of anthropogenic transformation of the ground: a case study of the Warsaw environs, Poland. Eng Geol.

[CR48] Krinitzsky EL, Hynes ME, Franklin AG (1997). Earthquake safety evaluation of sanitary landfills. Eng Geol.

[CR49] Lanz E, Maurer H, Green AG (1998). Refraction tomography over a buried waste disposal site. Geophysics.

[CR50] Loke MH, Barker RD (1996). Least-squares deconvolution of apparent resistivity pseudosections. Geophysics.

[CR51] Loke MH (1997) Electrical imaging surveys for environmental and engineering studies, a practical guide to 2-D and 3-D surveys: RES2DINV and RES2MOD Manual, 11700 Penang, Malaysia

[CR52] Loke MH (2004) Tutorial: 2-D and 3-D electrical imaging surveys. Geotomo Software, RES2DINV 3.5 Software, Malaysia

[CR53] McKenzie D (1972). Active tectonics of the Mediterranean region. Geophys J R Astr Soc.

[CR54] Meisner A, Krylov O, Nemlock M (2009) Development and structural architecture of the Eastern Black Sea, The Leading Edge 30: 1046–1055

[CR55] Midorikawa S (1987). Prediction of iso-seismal map in the Kanto Plain due to hypothetical earthquake. J Struct Eng.

[CR56] Mondelli M, Giacheti GL, Elis VR (2012) Geo-environmental site investigation for municipal solid waste disposal sites, in municipal and industrial waste disposal. London, United Kingdom: IntechOpen, 2012: https://www.intechopen.com/chapters/35153. 10.5772/28835

[CR57] Neyamadpour A (2019) 3D monitoring of volumetric water content using electrical resistivity tomography in municipal solid waste landfill. Environ Earth Sci 78. 10.1007/s12665-019-8436-4

[CR58] Nikishin AM, Korotaev MV, Ershov A, Brunet MF (2003). The Black Sea basin: tectonic history and Neogene-Quaternary rapid subsidence modeling. Sed Geol.

[CR59] Okay AI, Sahintürk Ö (1997) Geology of the eastern Pontides. In A.G. Robinson (Ed.), Regional and petroleum geology of the black sea and surrounding region. American Association of Petroleum Geologists Memoir 68: 291–311

[CR60] Olona J, Pulgar JA, Fernández-Viejo G, López-Fernández C, González-Cortina JM (2010). Weathering variations in a granitic massif and related geotechnical properties through seismic andelectrical resistivity methods. Near Surf Geophys.

[CR61] Önal AÖ, Demirbilek D, Demir V (2013). Geo-environmental site investigation for Tunceli, Turkey municipal solid waste disposal site. Eng Geol.

[CR62] Otto JC, Sass O (2006). Comparing geophysical methods for talus slope investigations in the Turtmann valley (Swiss Alps). Geomorphology.

[CR63] Özel S (2018). An evaluation on the sustainable environmental protection and environmental impact of disposal areas in Turkey. Eur J Sci Technol.

[CR64] Özel S, Yılmaz A, Candansayar ME (2017). The examination of the spread of the leachates coming out of a solid waste disposal area on the ground with geophysical and geochemical methods (Sivas, Turkey). J Appl Geophys.

[CR65] Öztürk S (2012). Statistical correlation between b value and fractal dimension regarding Turkish epicentre distribution. Earth Sci Res J.

[CR66] Papazachos BC (1999). An alternative method for a reliable estimation of seismicity with an application in Greece and the surrounding area. Bull Seismo Soc.

[CR67] Park CB, Miller RD, Xia J (1999). Multichannel analysis of surface waves. Geophysics.

[CR68] Pomposiello C, Dapena C, Favetto A, Boujon P (2012) Application of geophysical methods to waste disposal studies, in Municipal and Industrial Waste Disposal. London, United Kingdom: IntechOpen, https://www.intechopen.com/chapters/35152. 10.5772/29615

[CR69] Rao KS (1997). Site selection for a landfill.

[CR70] Reynolds JM, Taylor DI (1996) The use of geophysical surveys during the planning, construction and remediation of landfills, In: Bentley SP (Ed.), Engineering Geology of Waste Disposal and Storage, Geological Society Engineering Geology Special Publication. pp 93–98. 10.1144/GSL.ENG.1996.011.01.11

[CR71] Sayıl N (2014). Evaluation of the seismicity for the Marmara region with statistical approaches. Acta Geodaet Et Geophys.

[CR72] Schrott L, Sass O (2008). Application of field geophysics in geomorphology: advances and limitations exemplified by case studies. Geomorphology.

[CR73] SeisImager/SW (2022) Manual V 1.4 WindowsTM software for analysis of surface waves (V. 7.6), including explanation of geometrics seismodule controller software surface wave data acquisition wizards. ftp://geom.geometrics.com/pub/seismic/SeisImager. Accessed October 2022

[CR74] SeisImager/2D (2009) Manual V 3.3 WindowsTM Software for Pickwin (V. 4.0.1.5) and Plotrefa (V. 2.9.1.6), https://geometrics.com/wp-content/uploads/2019/05/SeisImager2D_Manual_v3.3.pdf. Accessed Oct 2022

[CR75] Silvestri S, Omri M (2008). A method for the remote sensing identification of uncontrolled landfills: formulation and validation. Int J Remote Sens.

[CR76] Siracusa G, La Rosa AD, Giuffrida G, Leotta A (2005). Geochemical and geophysical characterisation of a municipal solid waste landfill. Ecosyst Sustain Dev.

[CR77] Smith DL, Randazzo AF (2003). Application of electrical resistivity measurements to an evaluation of a potential landfill site in a karstic terrain. Environ Geol.

[CR78] Socco LV, Strobbia C (2004). Surface -wave method for near -surface characterization: a tutorial. Near Surf Geophys.

[CR79] Soupios P, Papadopoulos I, Kouli M, Georgaki I, Vallianatos F, Kokkinou E (2007). Investigation of waste disposal areas using electrical methods: a case study from Chania, Crete, Greece. Environ Geol.

[CR80] Soupios P, Ntarlagiannis D (2017) Characterization and monitoring of solid waste disposal sites using geophysical methods: current applications and novel trends. In: Modelling Trends in Solid and Hazardous Waste Management. Springer Singapore 75–103. 10.1007/978-981-10-2410-8_5

[CR81] TBEC (Türkiye Building Earthquake Code) (2018) Türkiye bina ve deprem yönetmeliği, T.C. Resmi Gazete, Çevre Şehircilik ve İklim Bakanlığı, Ankara, Türkiye (in Turkish)

[CR82] Tchobanoglous G, Theisen H, Vigil SA (1993). Integrated solid waste management engineering principles management issues.

[CR83] Vargemezis G, Tsourlos P, Giannopoulos A, Trilyrakis P (2015). 3D electrical resistivity tomography technique for the investigation of a construction and demolition waste landfill site. Stud Geophys Geod.

[CR84] Wiemer S, Wyss M (1997). Mapping the frequency-magnitude distributions in asperities: an improved technique to calculate recurrence times. J Geopyhys Res.

[CR85] Yeşilnaçar Mİ, Çetin H (2005). Site selection for hazardous wastes: a case study from the GAP area, Turkey. Eng Geol.

[CR86] Yildirim V, Uzun B, Baykal MT, Terzi F, Atasoy BA (2022). Odor-aided analysis for landfill site selection: study of DOKAP Region, Turkey. Environ Sci Pollut Res.

[CR87] Zhan TLT, Chen YM, Lin WA (2008). Shear strength characterization of municipal solid waste at the Suzhou landfill, China. Eng Geol.

[CR88] Zhan LT, Zhang Z, Chen YM, Chen R, Zhang S, Liu J, Li AG (2018). The 2015 Shenzhen catastrophic landslide in a construction waste dump: reconstitution of dump structure and failure mechanisms via geotechnical investigations. Eng Geol.

